# Supplementary material for: Proneurotrophin-3 contributes to chemotherapy-induced neuropathic pain through TrkC-mediated CCL2 elevation in DRG neurons
Source: EMBO Rep. 2025 Nov 26;26(24):6141–58. doi: 10.1038/s44319-025-00534-1 (PMC12714783; doi:10.1038/s44319-025-00534-1)
Supplement: Supplementary file 1 — Table EV1 [file 44319_2025_534_MOESM1_ESM.pdf]

**Table EV 1**: Locomotor function.

| Treatment groups | Placing | Grasping | Righting |
|---|---|---|---|
| Scr + Veh (male) | 5 (0) | 5 (0) | 5 (0) |
| Scr + PTX (male) | 5 (0) | 5 (0) | 5 (0) |
| Si + PTX (male) | 5 (0) | 5 (0) | 5 (0) |
| Si + Veh (male) | 5 (0) | 5 (0) | 5 (0) |
| Scr + Veh (female) | 5 (0) | 5 (0) | 5 (0) |
| Scr + PTX (female) | 5 (0) | 5 (0) | 5 (0) |
| Si + PTX (female) | 5 (0) | 5 (0) | 5 (0) |
| Si + Veh (female) | 5 (0) | 5 (0) | 5 (0) |
| GFP (male) | 5 (0) | 5 (0) | 5 (0) |
| NT3 (male) | 5 (0) | 5 (0) | 5 (0) |
| GFP (female) | 5 (0) | 5 (0) | 5 (0) |
| NT3 (female) | 5 (0) | 5 (0) | 5 (0) |
| Scr + GFP (male) | 5 (0) | 5 (0) | 5 (0) |
| Scr + NT3 (male) | 5 (0) | 5 (0) | 5 (0) |
| Si-T + GFP (male) | 5 (0) | 5 (0) | 5 (0) |
| Si-T + NT3 (male) | 5 (0) | 5 (0) | 5 (0) |
| Si-P + GFP (male) | 5 (0) | 5 (0) | 5 (0) |
| Si-P + NT3 (male) | 5 (0) | 5 (0) | 5 (0) |
| $NT3^{f/f}$ + Veh (male) | 5 (0) | 5 (0) | 5 (0) |
| $NT3^{f/f}$ + PTX (male) | 5 (0) | 5 (0) | 5 (0) |
| NT3KD + Veh (male) | 5 (0) | 5 (0) | 5 (0) |
| NT3KD + PTX (male) | 5 (0) | 5 (0) | 5 (0) |

n = 9-12 mice per group; 5 trials/reflex/group; Mean (SD). NT3: AAV5-*NT3*. GFP:/AAV5-*Gfp*. Scr: Scrambled siRNA. Si: NT3 siRNA. Si-T: Trkc siRNA. Si-P: $P^{75}$NTR siRNA. $NT3^{f/f}$: NT3 flox. NT3KD: NT3 conditional knockdown. Veh: Vehicle. PTX: Paclitaxel.
