# Supplementary material for: Proneurotrophin-3 contributes to chemotherapy-induced neuropathic pain through TrkC-mediated CCL2 elevation in DRG neurons
Source: EMBO Rep. 2025 Nov 26;26(24):6141–58. doi: 10.1038/s44319-025-00534-1 (PMC12714783; doi:10.1038/s44319-025-00534-1)
Supplement: Supplementary file 2 — Table EV2 [file 44319_2025_534_MOESM2_ESM.pdf]

Table EV 2. Primers used

Gene Name	Forward Primer	Reverse Primer
RT-PCR		
Nt3	5’- ACCACGGAGGAAACGCTATG -3’	5’- CCCCGAATGTCAATGGCTGA -3’
Ccl2	5’- CCACTCACCTGCTGCTACTC -3’	5’- GCTGCTGGTGATCCTCTTGT -3’
TrkC	5’- CATTGCAGAGAACGTGGTGG -3’	5’- CCCTCCTGGTAGTAGTCCATGT-3’
P75	5’- GGATCACAAGGTCTACGCCC -3’	5’- GGAATGAGGTTGTCAGCGGT -3’
Construction		
Nt3	5’-GGCCTCGAGATGTCCATCTTGTTTTATG-3’	5-CGCGCGGCCGCTCATGTTCTTCCAATTTTTC-3
