# Supplementary figures and images for: Proneurotrophin-3 contributes to chemotherapy-induced neuropathic pain through TrkC-mediated CCL2 elevation in DRG neurons
Source: EMBO Rep. 2025 Nov 26;26(24):6141–58. doi: 10.1038/s44319-025-00534-1 (PMC12714783; doi:10.1038/s44319-025-00534-1)

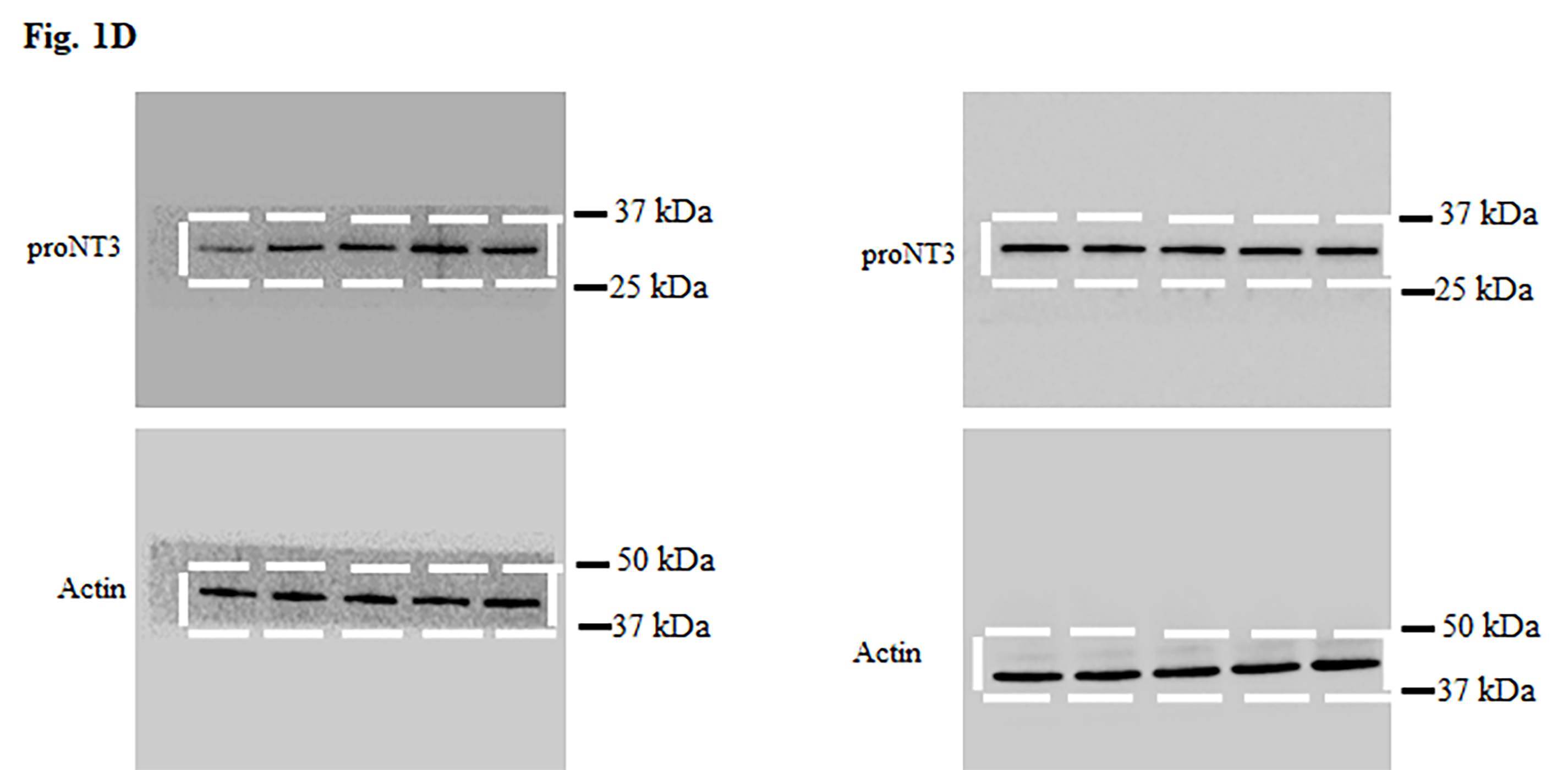

Supplement: Supplementary file 4 — Source data Fig. 1 [file 44319_2025_534_MOESM4_ESM.zip › Figure 1/Fig. 1D.tif]

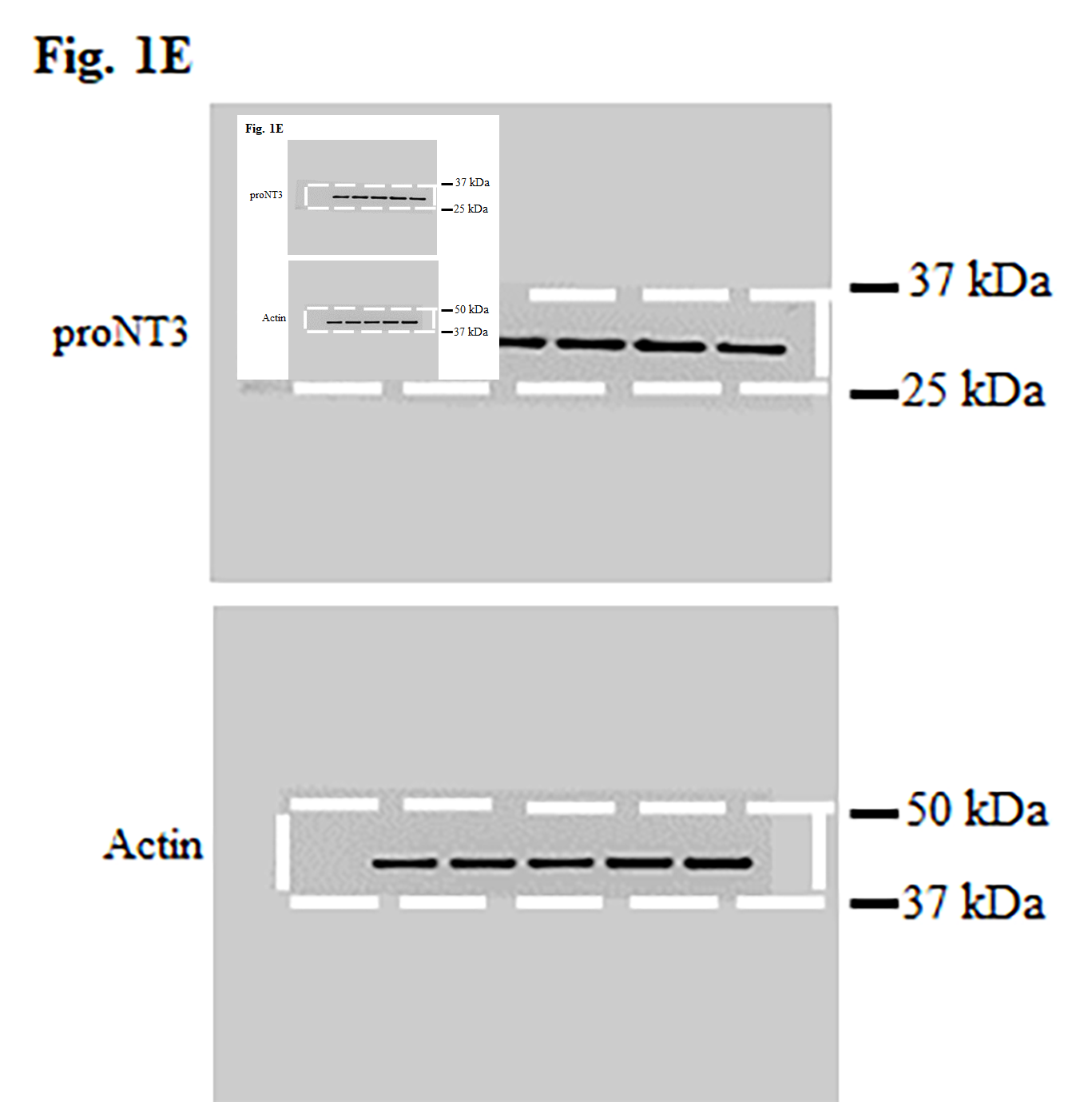

Supplement: Supplementary file 4 — Source data Fig. 1 [file 44319_2025_534_MOESM4_ESM.zip › Figure 1/Figure 1E.tif]

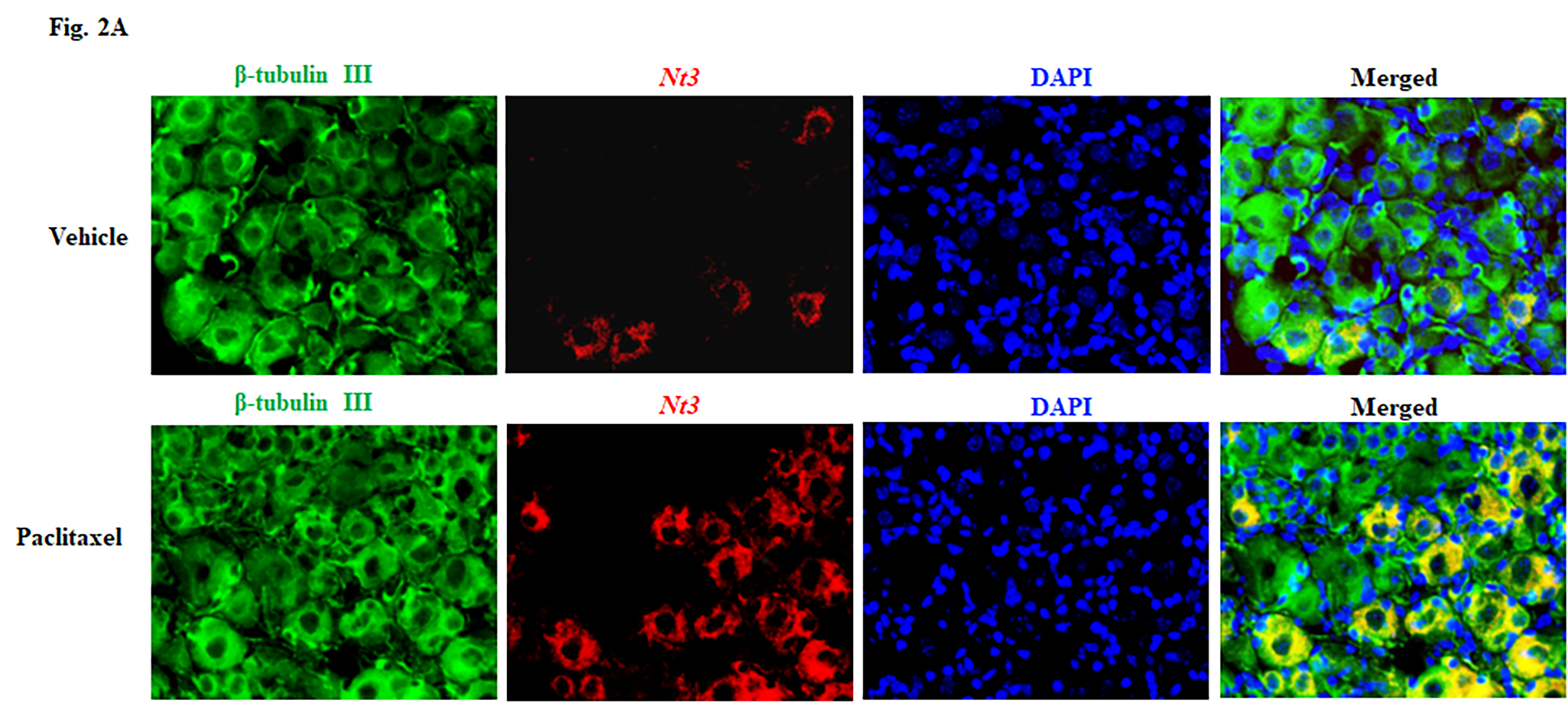

Supplement: Supplementary file 5 — Source data Fig. 2 [file 44319_2025_534_MOESM5_ESM.zip › Figure 2/2A.tif]

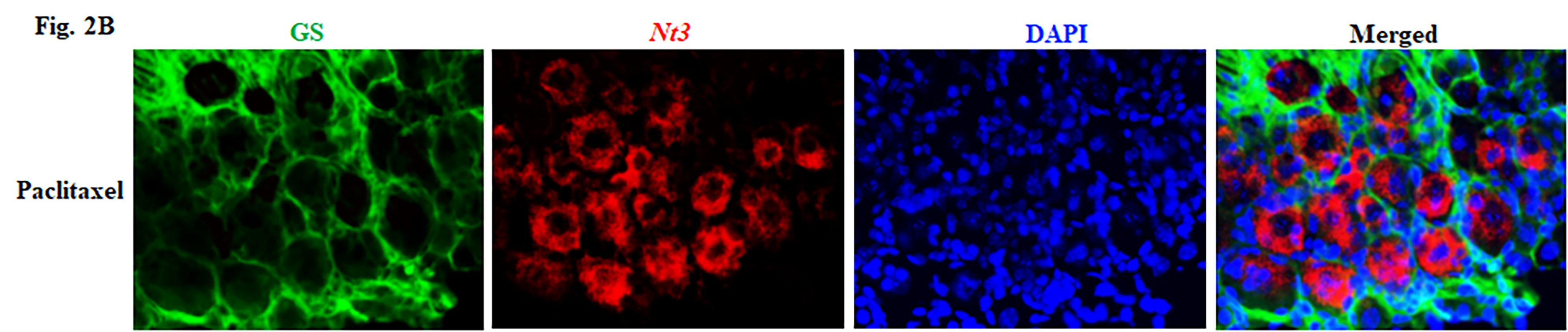

Supplement: Supplementary file 5 — Source data Fig. 2 [file 44319_2025_534_MOESM5_ESM.zip › Figure 2/2B.tif]

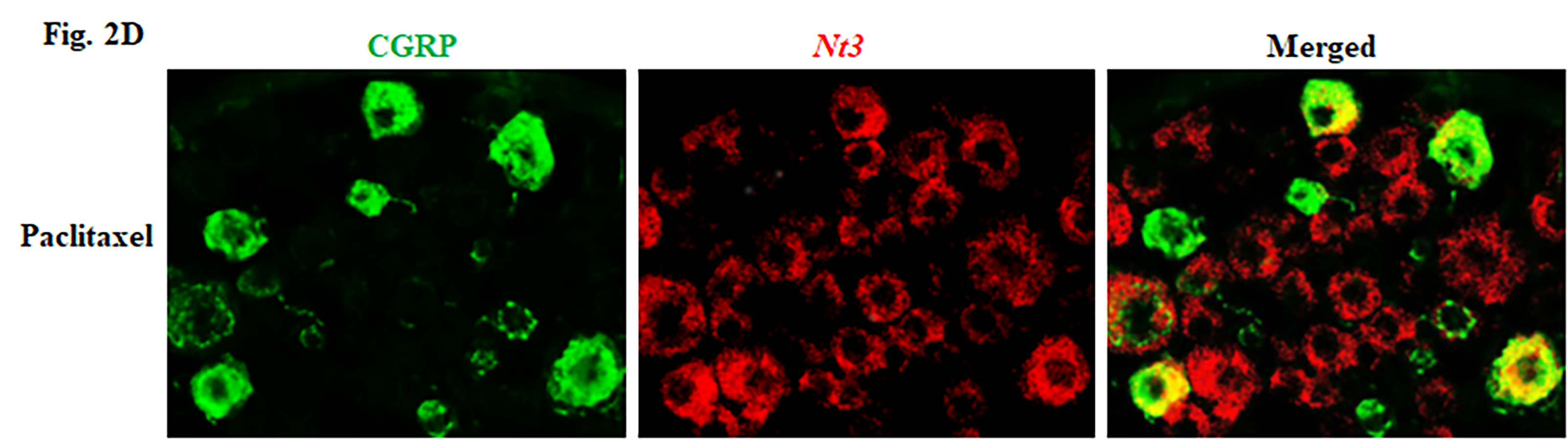

Supplement: Supplementary file 5 — Source data Fig. 2 [file 44319_2025_534_MOESM5_ESM.zip › Figure 2/2D.tif]

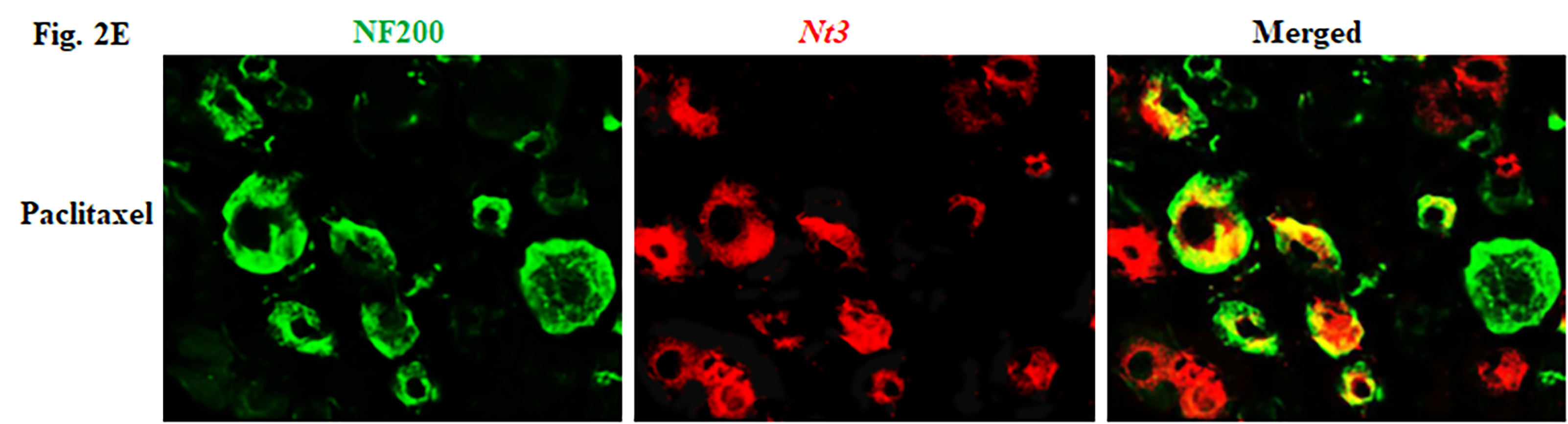

Supplement: Supplementary file 5 — Source data Fig. 2 [file 44319_2025_534_MOESM5_ESM.zip › Figure 2/2E.tif]

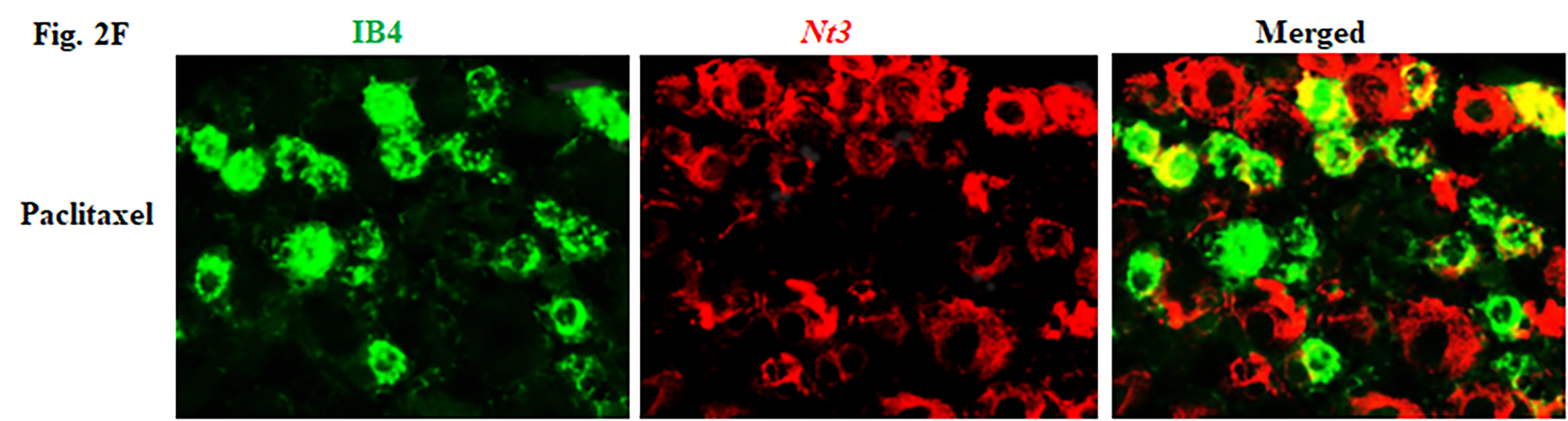

Supplement: Supplementary file 5 — Source data Fig. 2 [file 44319_2025_534_MOESM5_ESM.zip › Figure 2/2F.tif]

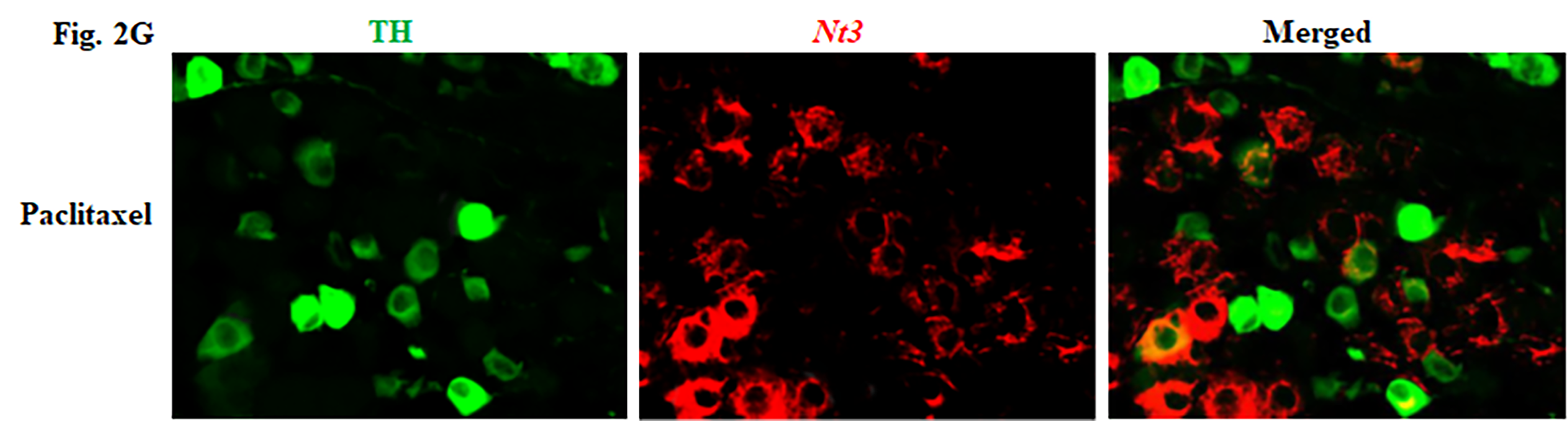

Supplement: Supplementary file 5 — Source data Fig. 2 [file 44319_2025_534_MOESM5_ESM.zip › Figure 2/2G.tif]

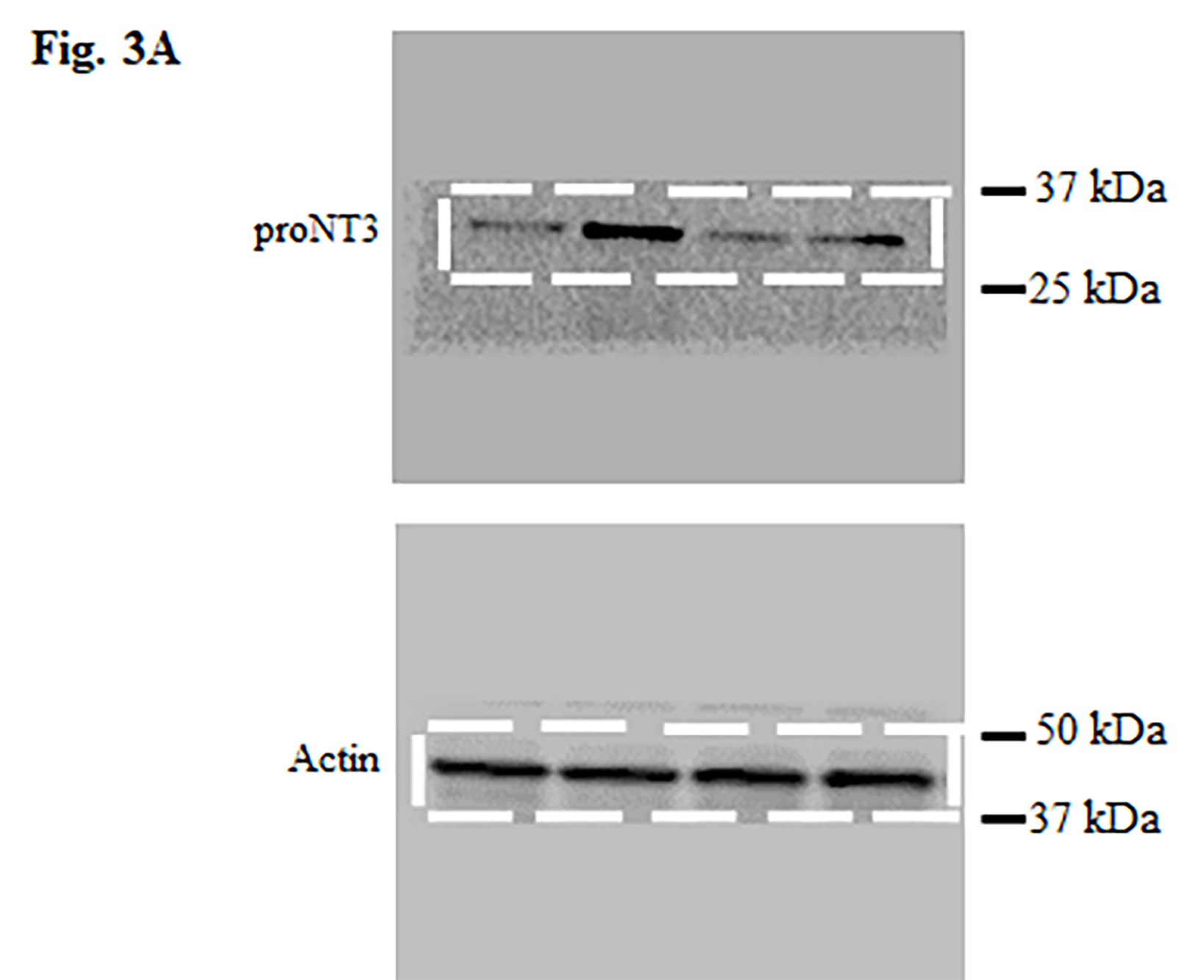

Supplement: Supplementary file 6 — Source data Fig. 3 [file 44319_2025_534_MOESM6_ESM.zip › Figure 3/3A.tif]

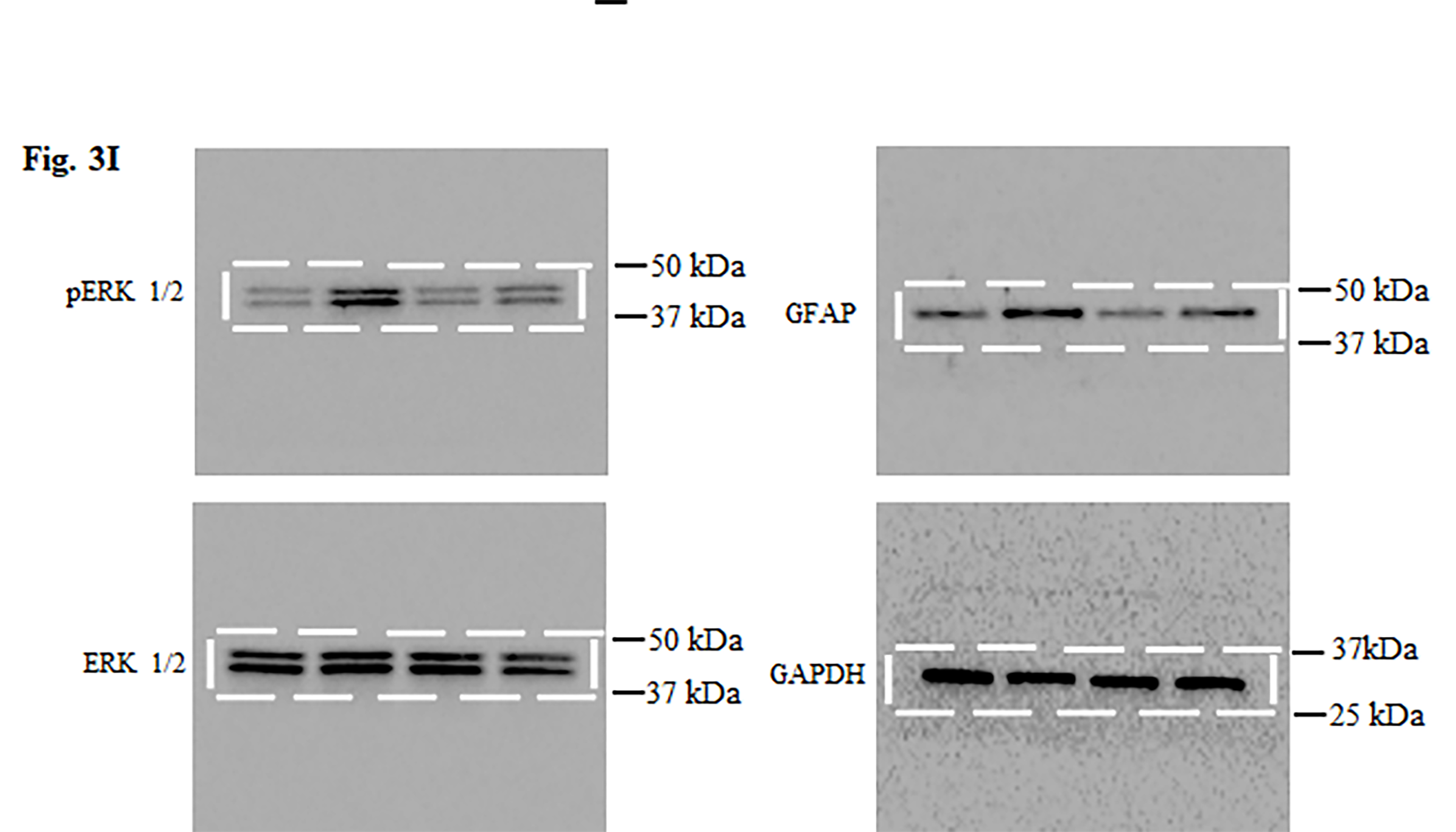

Supplement: Supplementary file 6 — Source data Fig. 3 [file 44319_2025_534_MOESM6_ESM.zip › Figure 3/3I.tif]

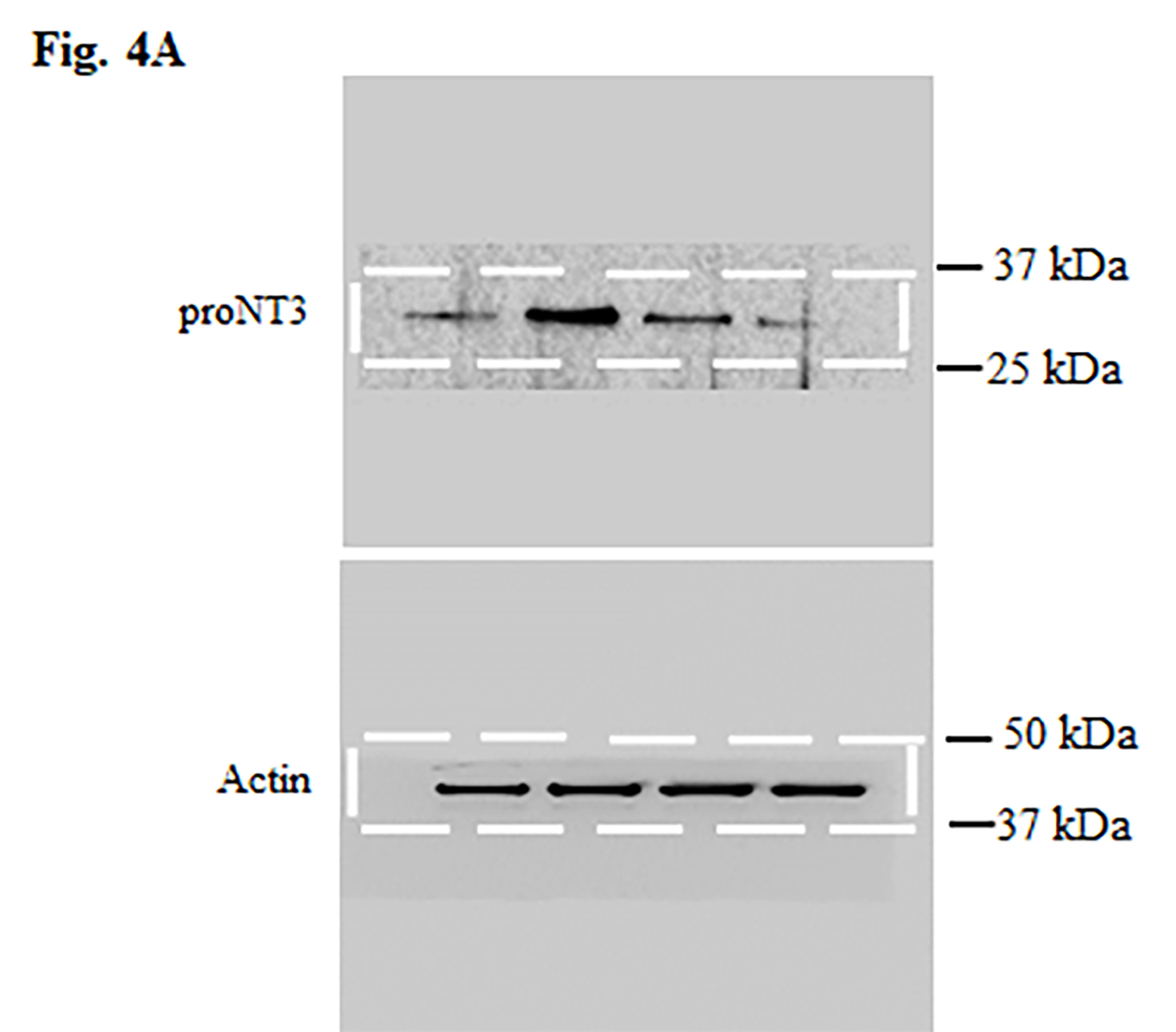

Supplement: Supplementary file 7 — Source data Fig. 4 [file 44319_2025_534_MOESM7_ESM.zip › Figure 4/4A.tif]

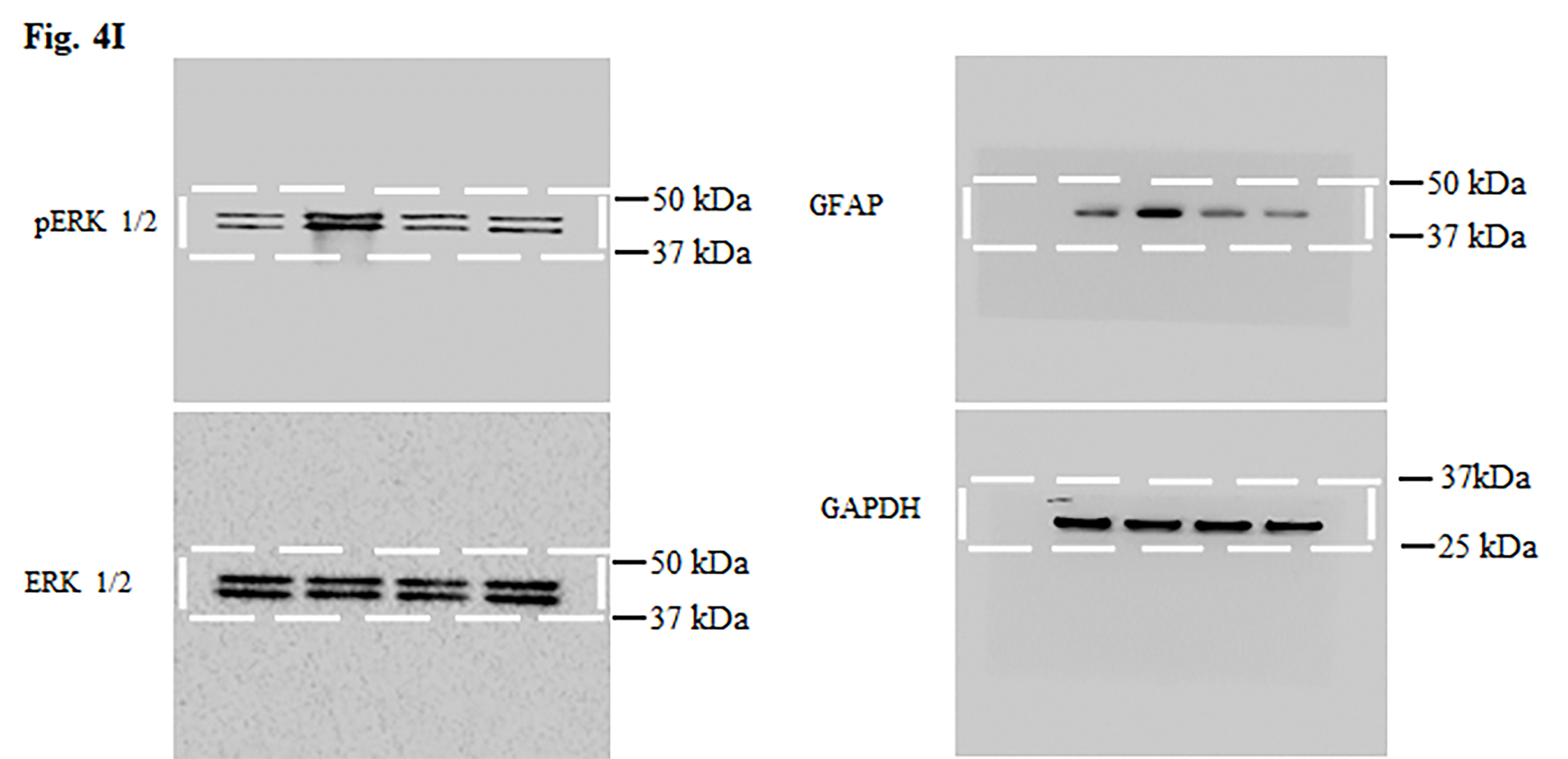

Supplement: Supplementary file 7 — Source data Fig. 4 [file 44319_2025_534_MOESM7_ESM.zip › Figure 4/4I.tif]

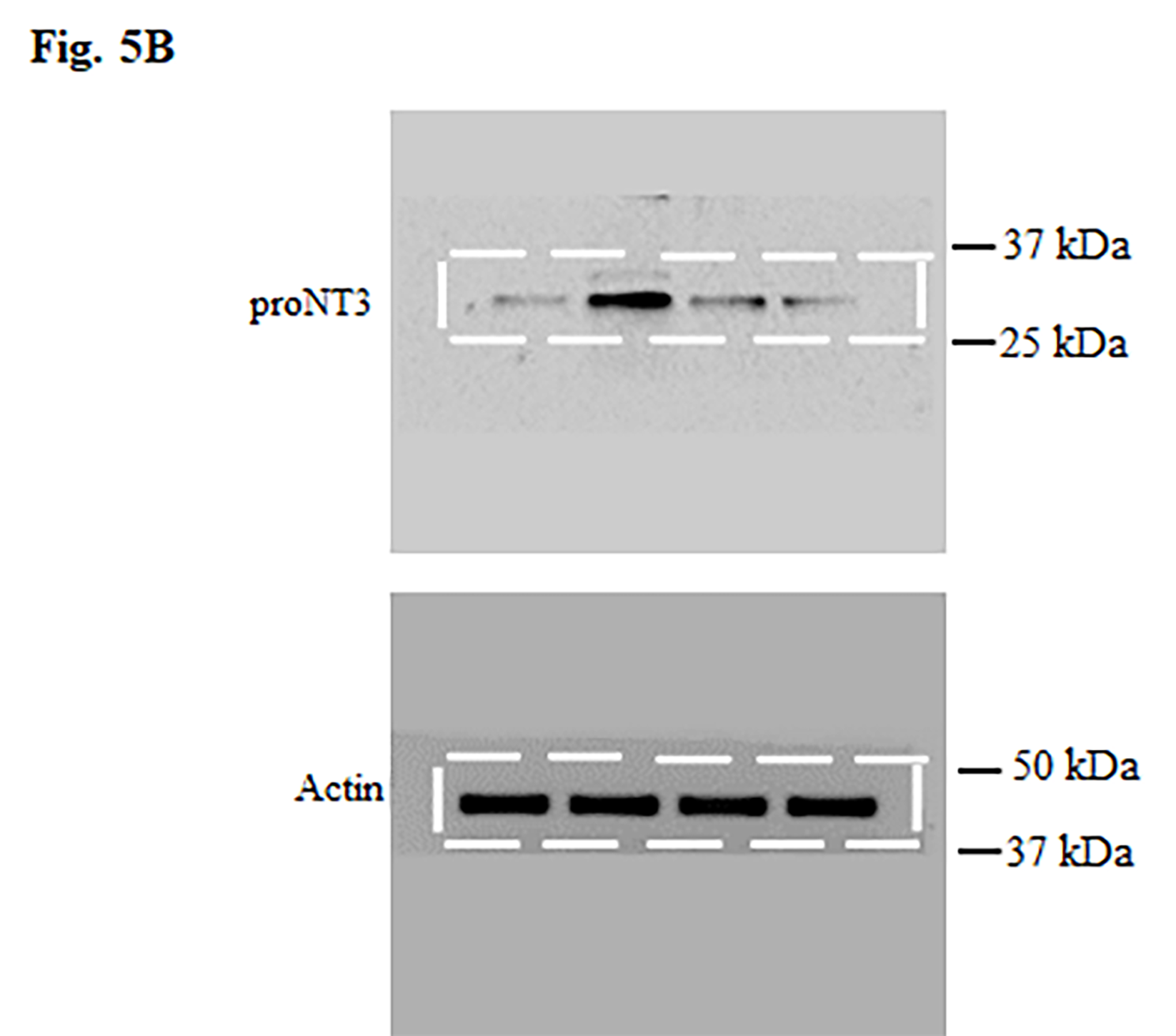

Supplement: Supplementary file 8 — Source data Fig. 5 [file 44319_2025_534_MOESM8_ESM.zip › Figure 5/5B.tif]

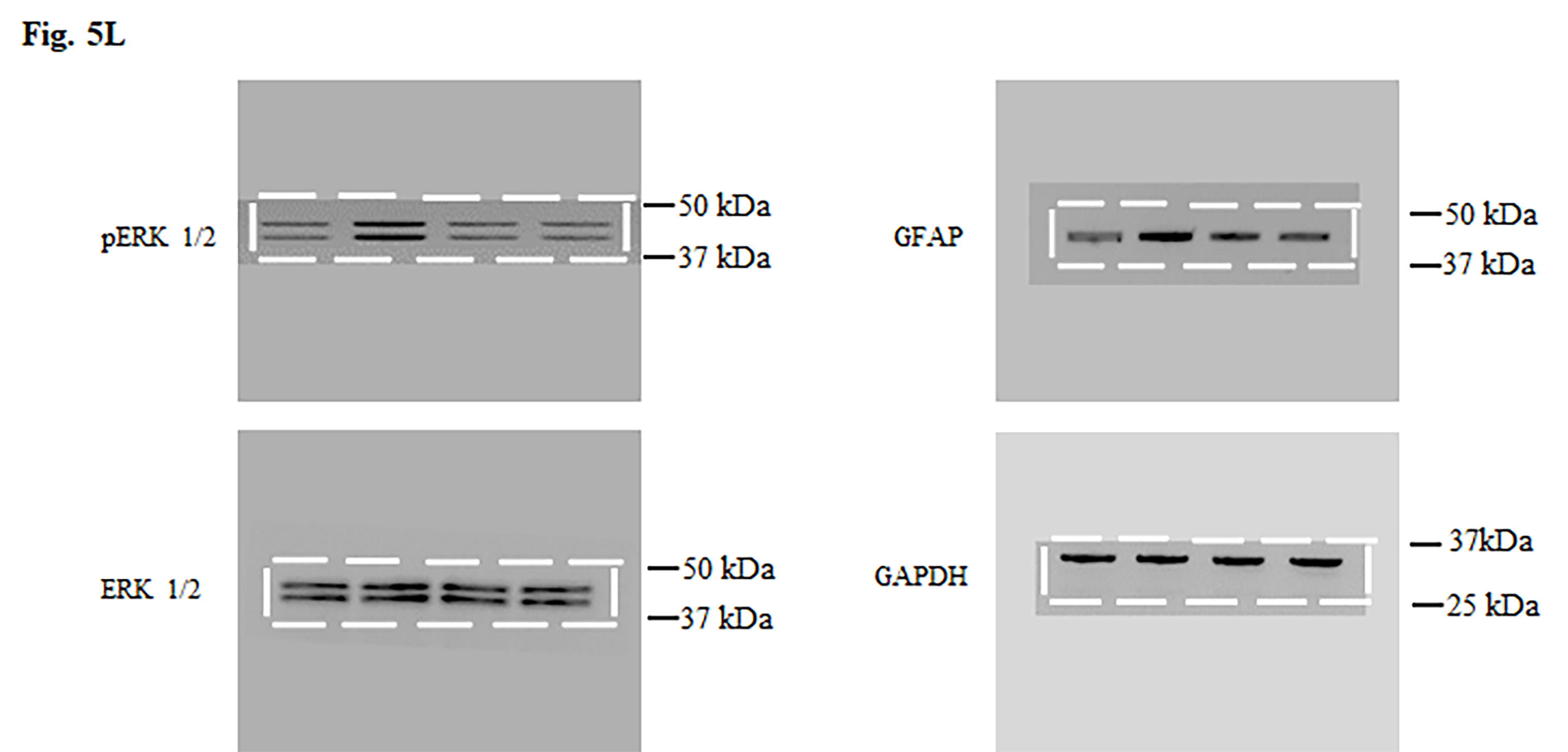

Supplement: Supplementary file 8 — Source data Fig. 5 [file 44319_2025_534_MOESM8_ESM.zip › Figure 5/5L.tif]

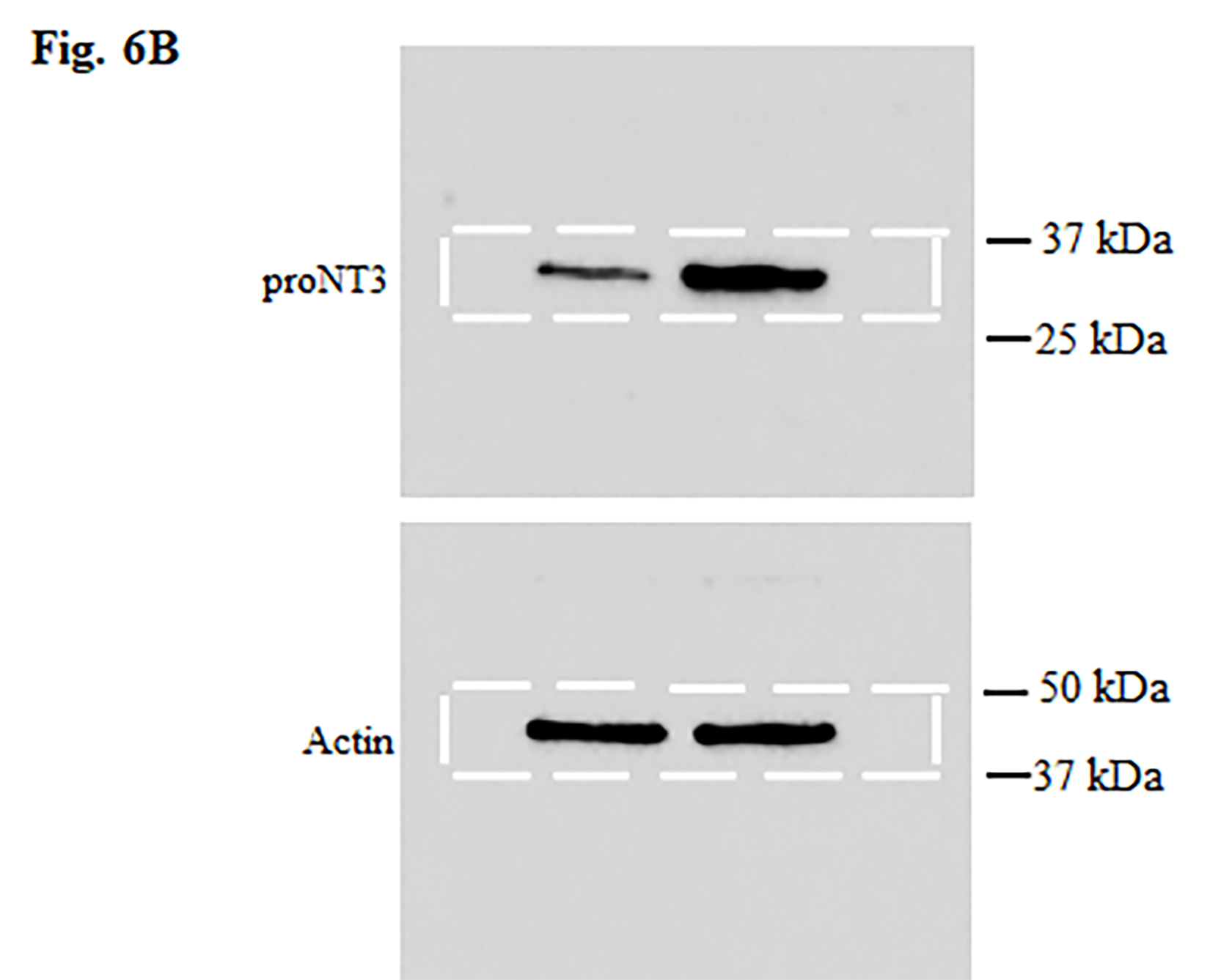

Supplement: Supplementary file 9 — Source data Fig. 6 [file 44319_2025_534_MOESM9_ESM.zip › Figure 6/6B.tif]

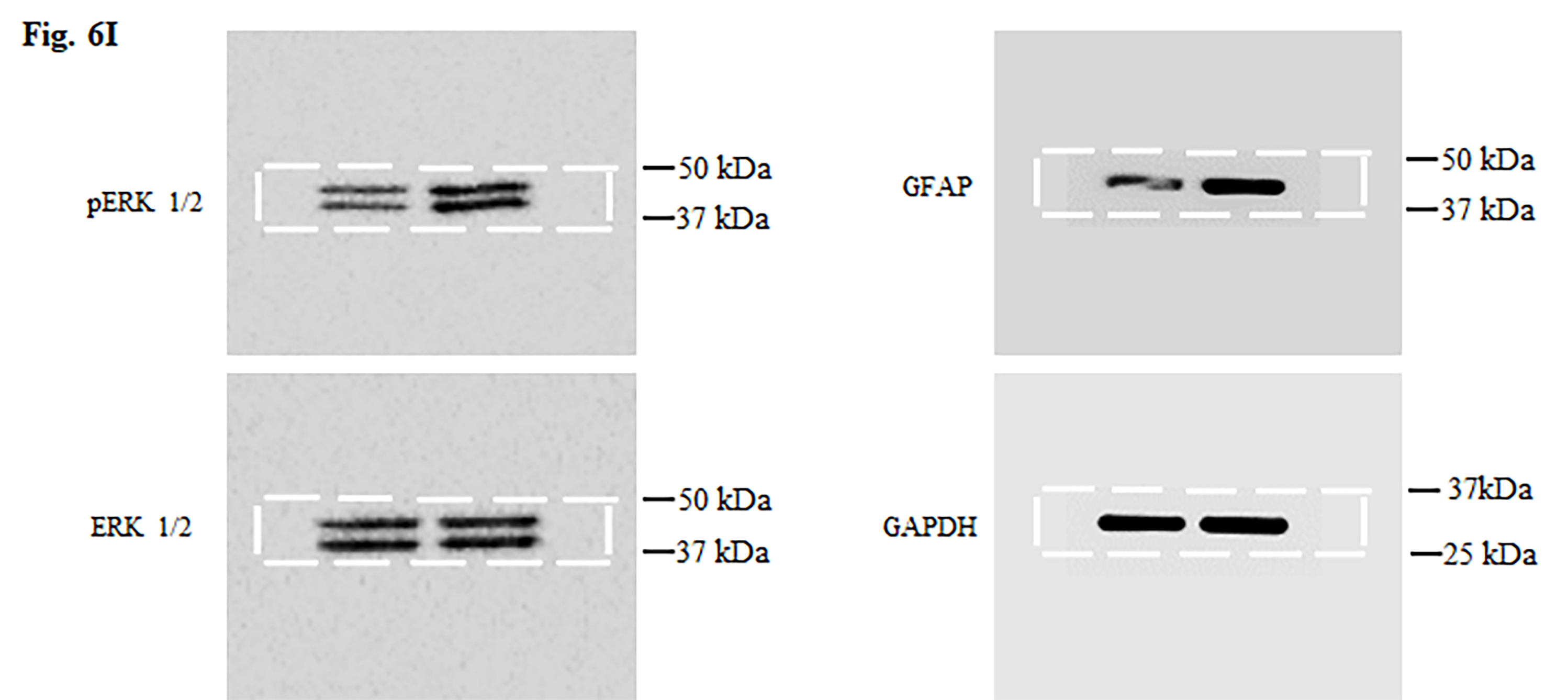

Supplement: Supplementary file 9 — Source data Fig. 6 [file 44319_2025_534_MOESM9_ESM.zip › Figure 6/6I.tif]

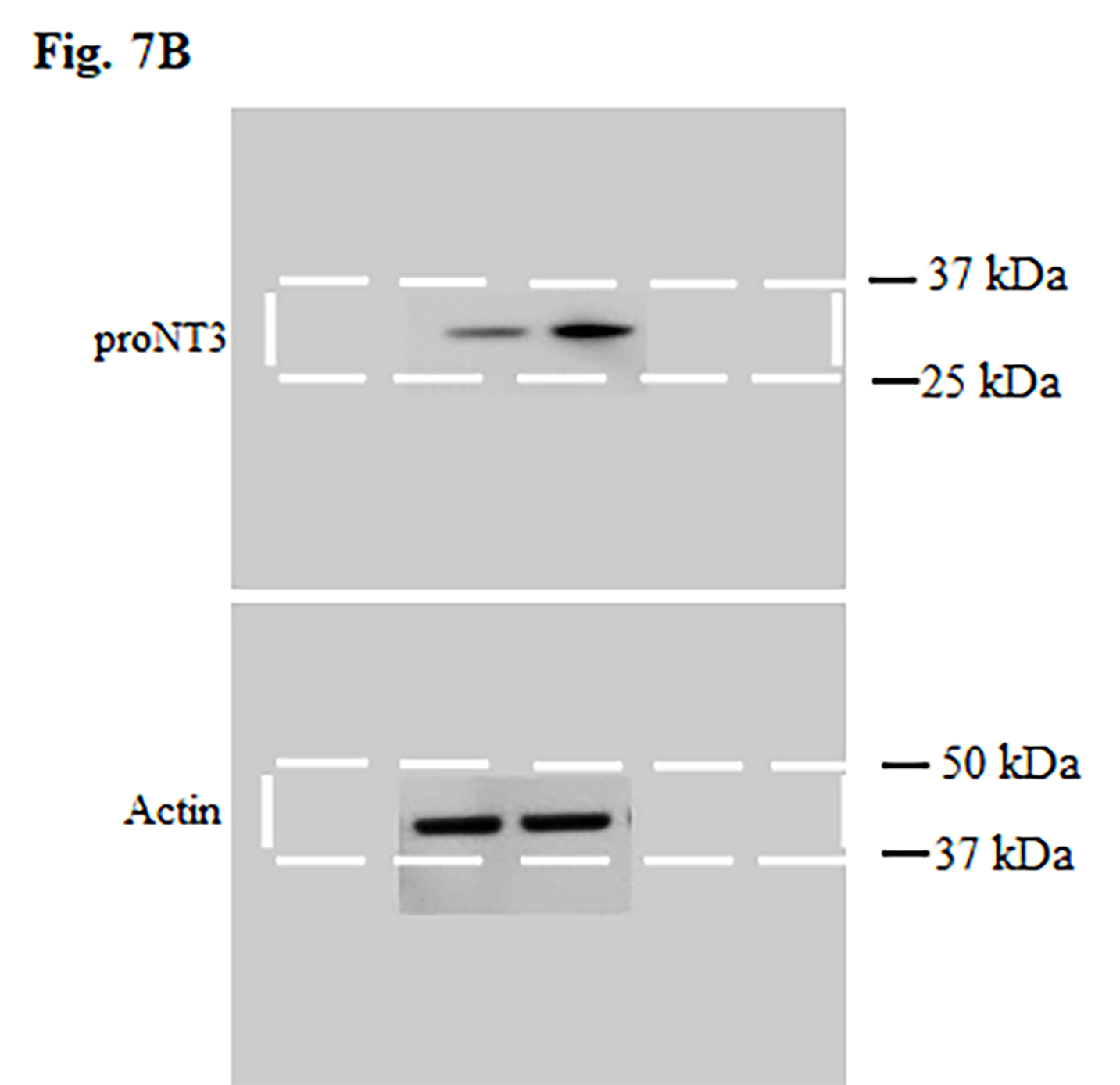

Supplement: Supplementary file 10 — Source data Fig. 7 [file 44319_2025_534_MOESM10_ESM.zip › Figure 7/7B.tif]

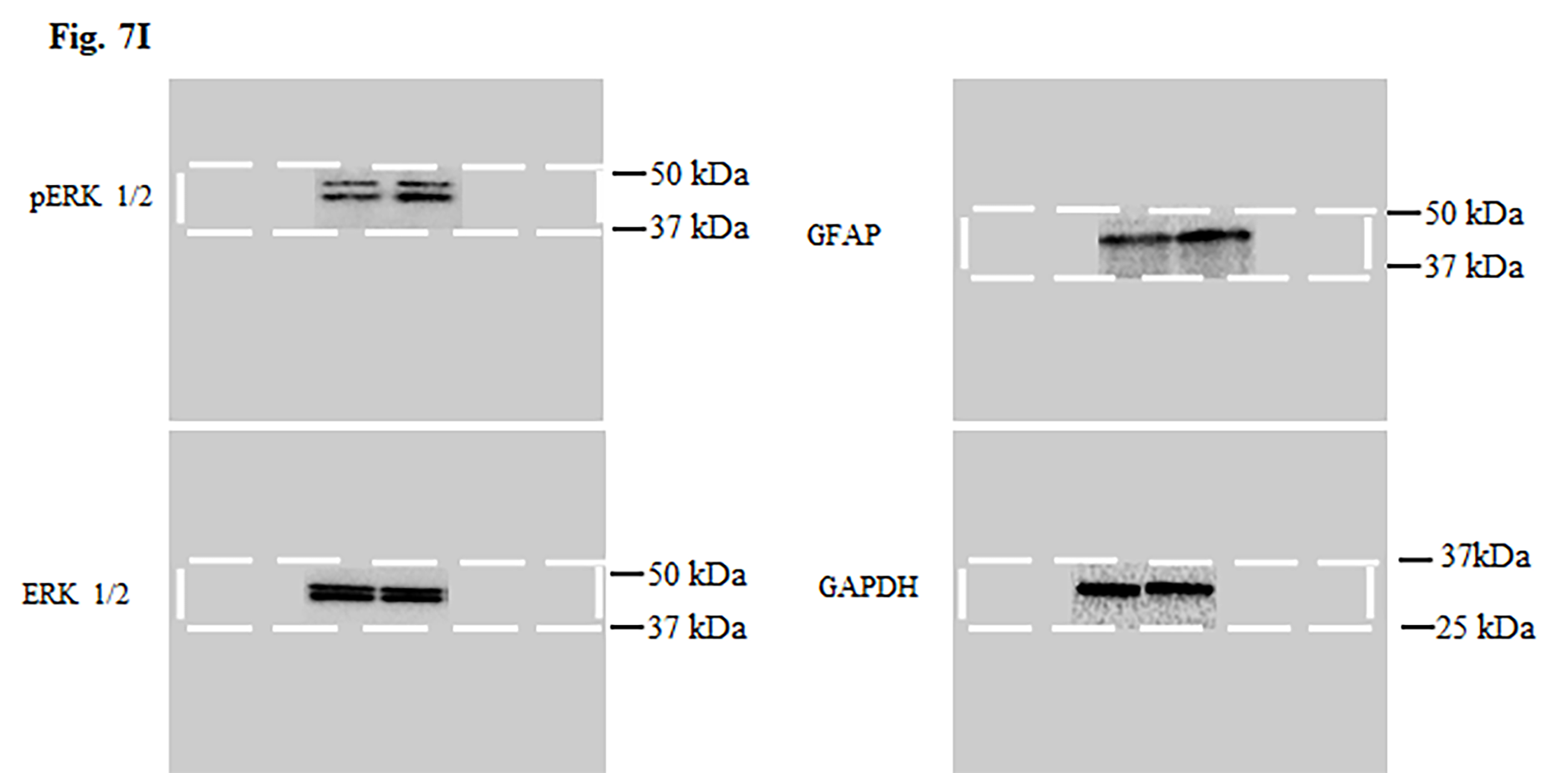

Supplement: Supplementary file 10 — Source data Fig. 7 [file 44319_2025_534_MOESM10_ESM.zip › Figure 7/7I.tif]

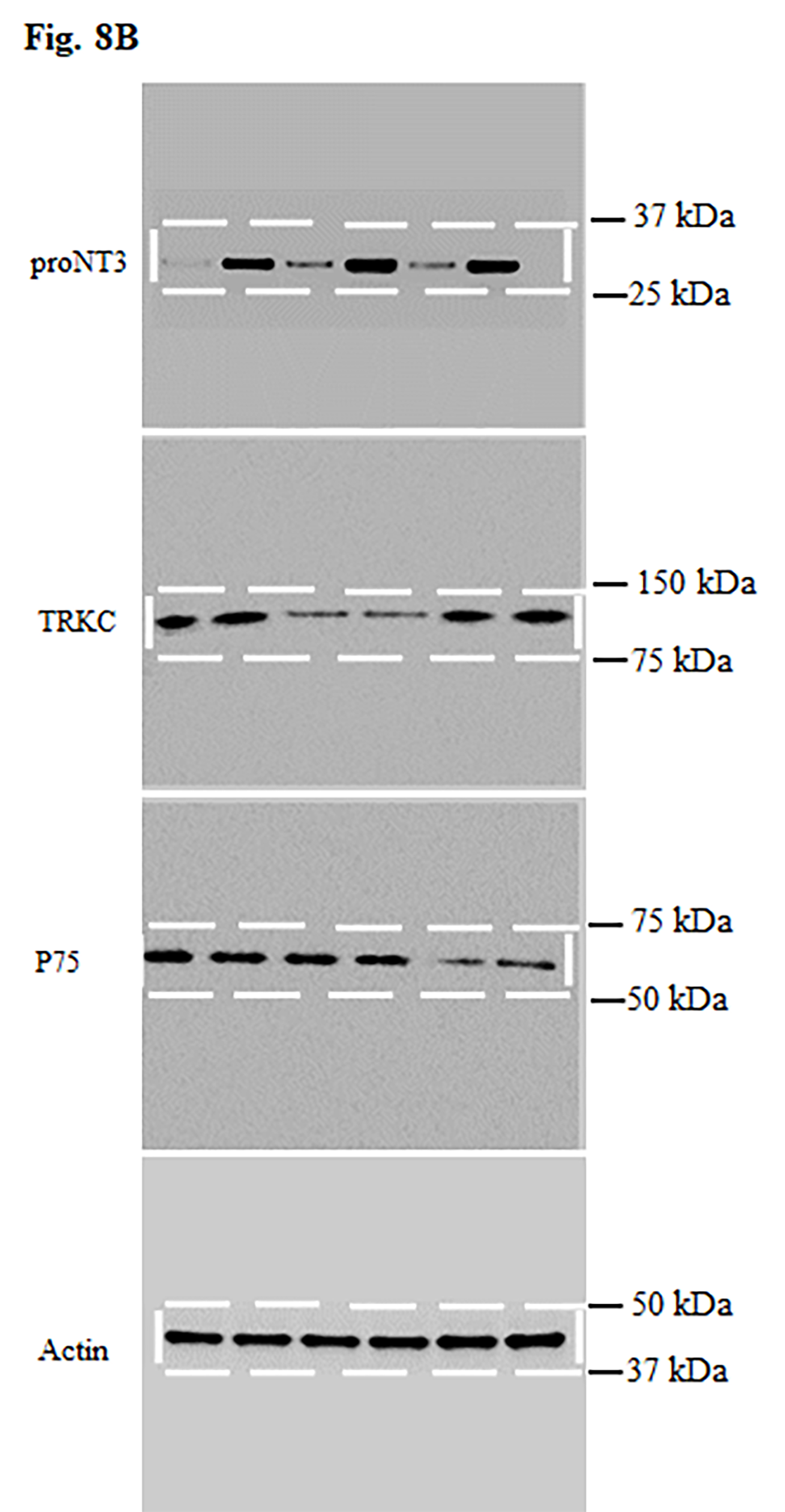

Supplement: Supplementary file 11 — Source data Fig. 8 [file 44319_2025_534_MOESM11_ESM.zip › Figure 8/8B.tif]

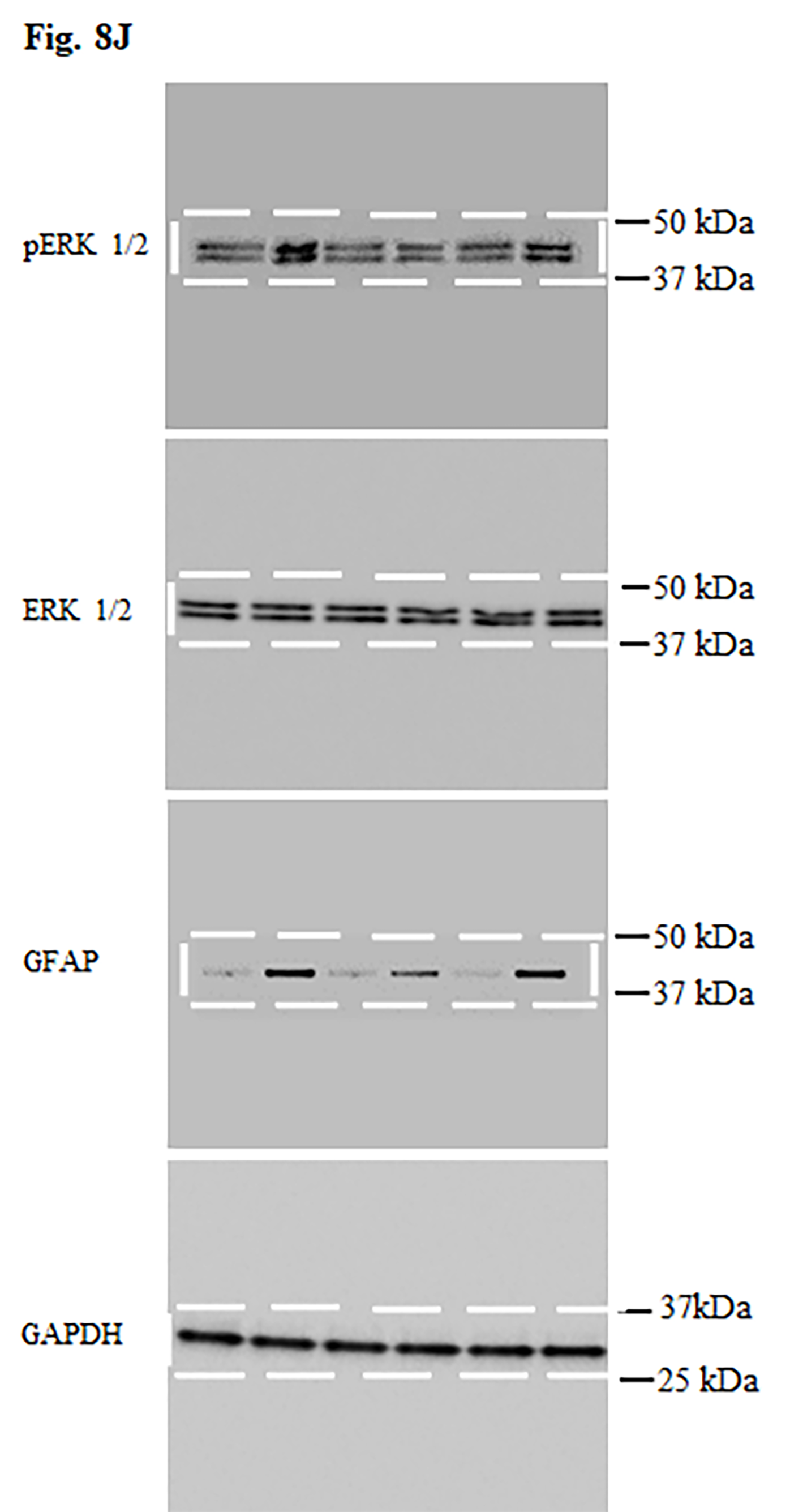

Supplement: Supplementary file 11 — Source data Fig. 8 [file 44319_2025_534_MOESM11_ESM.zip › Figure 8/8J.tif]

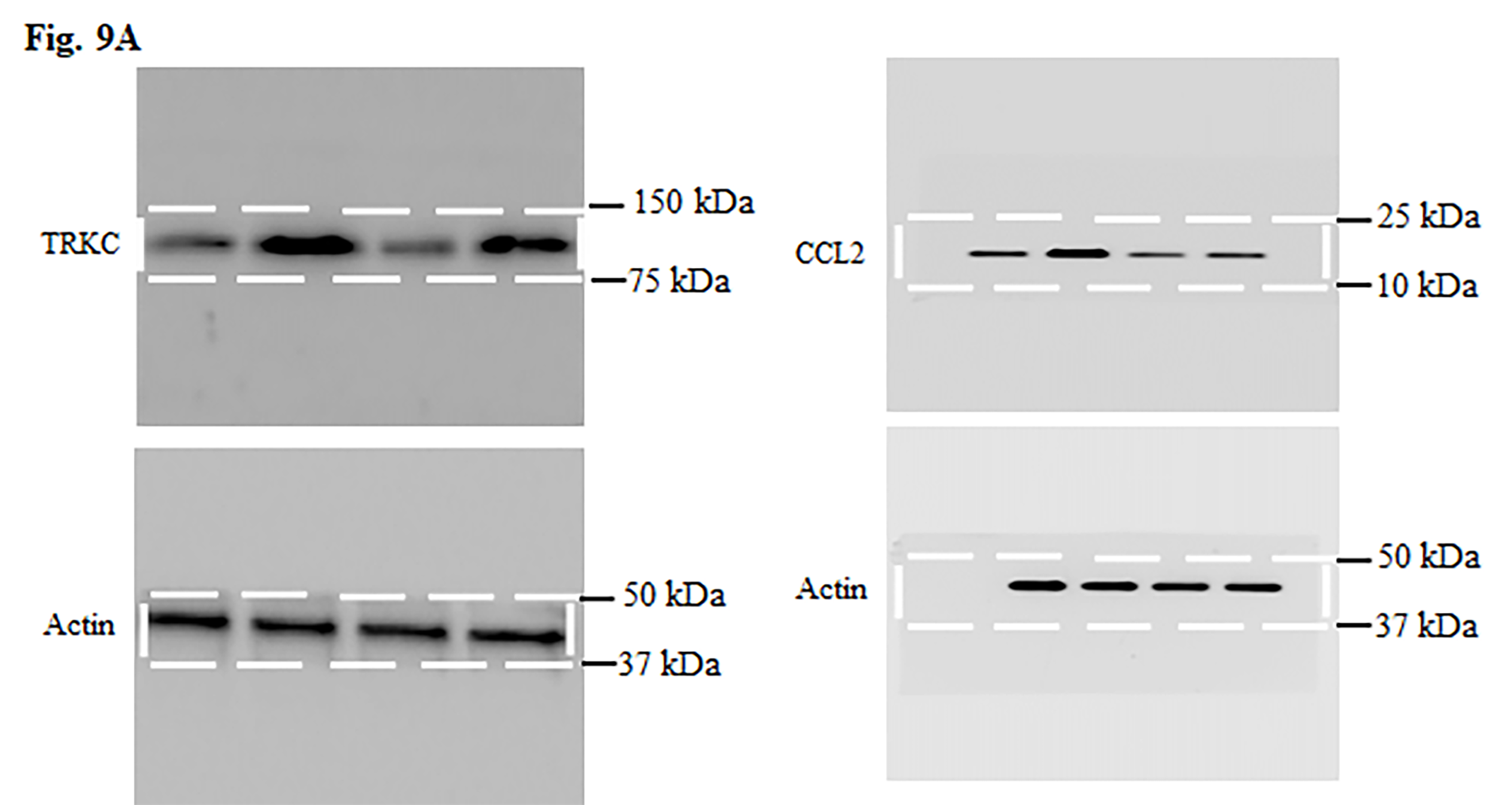

Supplement: Supplementary file 12 — Source data Fig. 9 [file 44319_2025_534_MOESM12_ESM.zip › Figure 9/9A.tif]

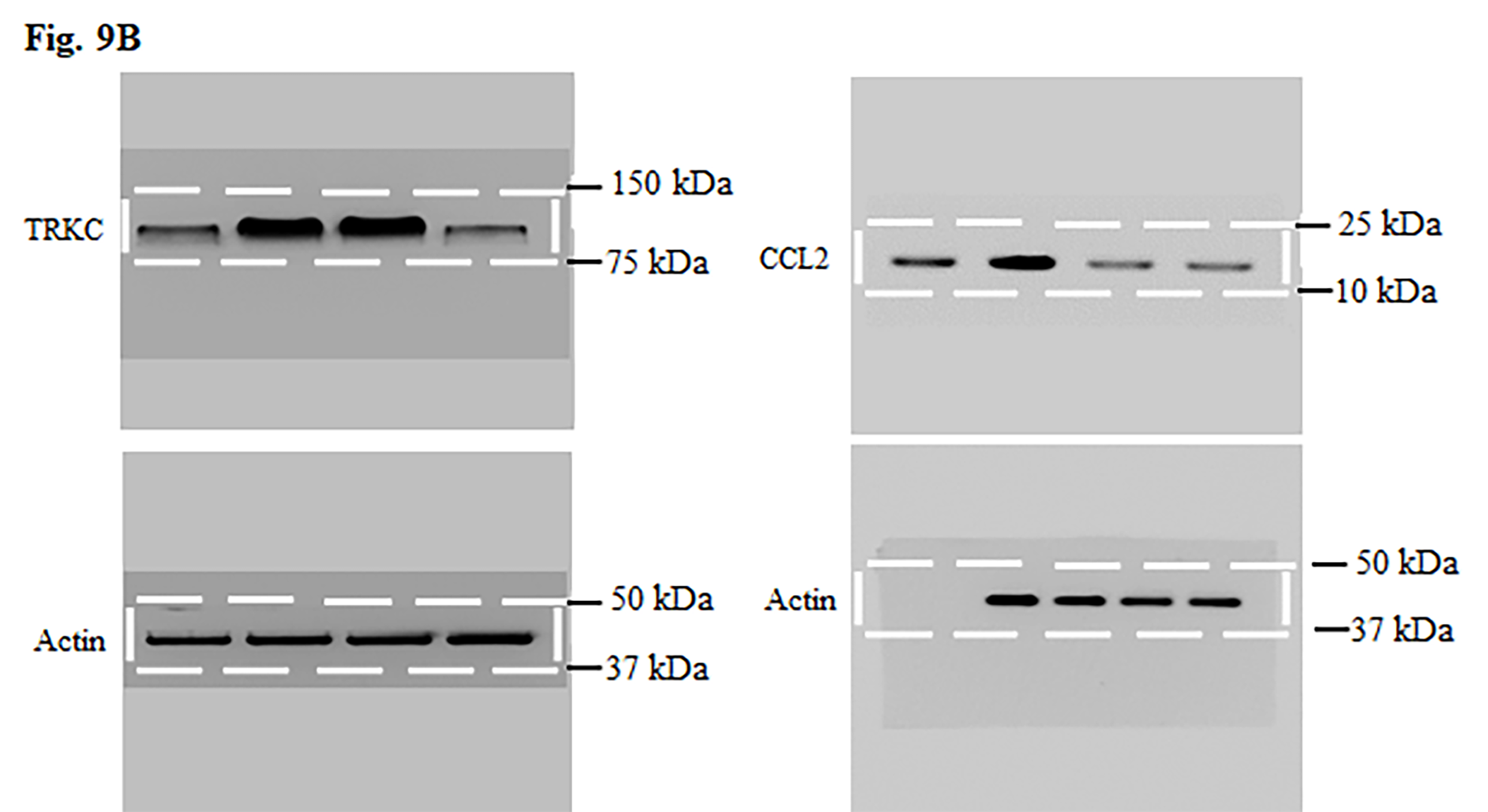

Supplement: Supplementary file 12 — Source data Fig. 9 [file 44319_2025_534_MOESM12_ESM.zip › Figure 9/9B.tif]

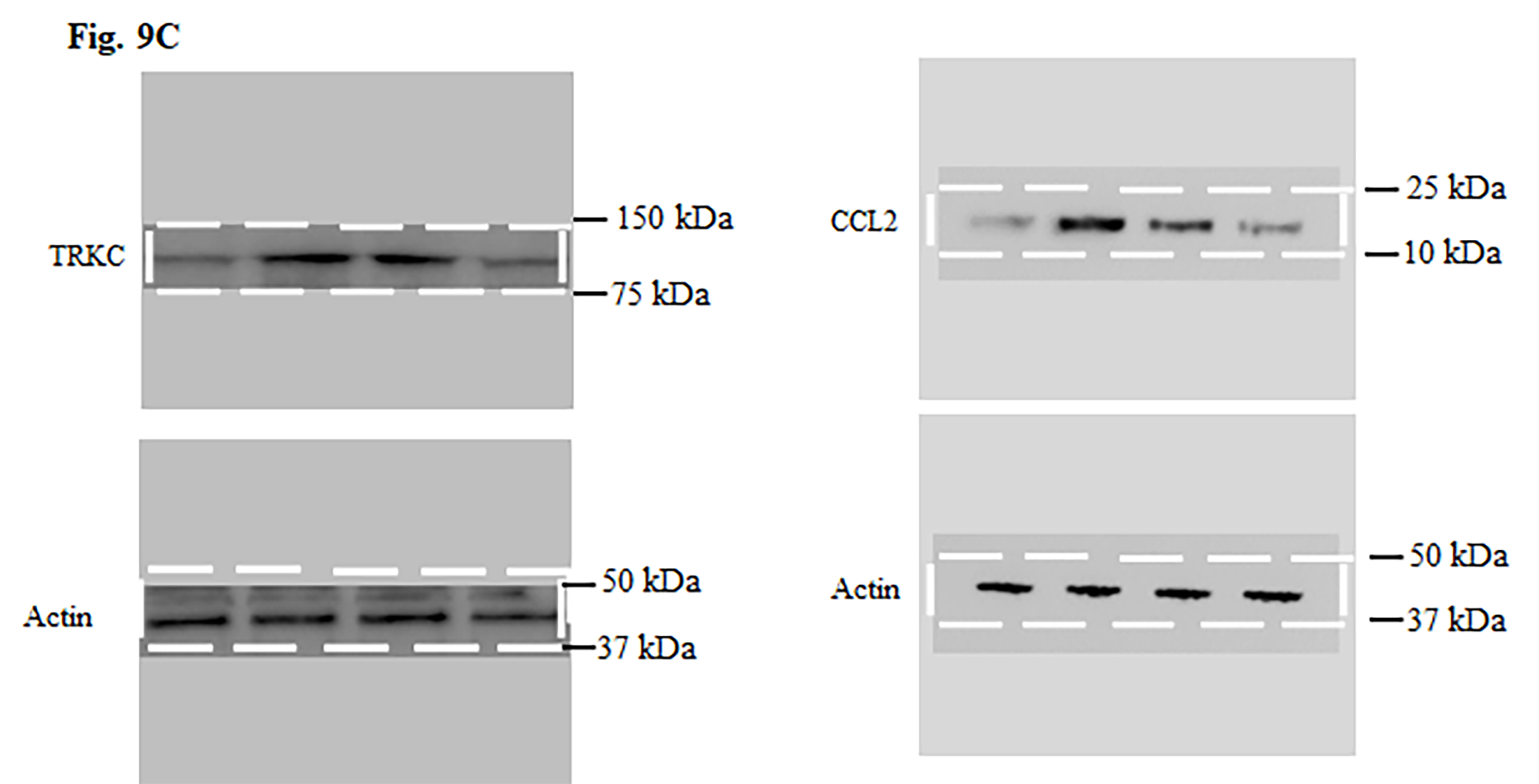

Supplement: Supplementary file 12 — Source data Fig. 9 [file 44319_2025_534_MOESM12_ESM.zip › Figure 9/9C.tif]

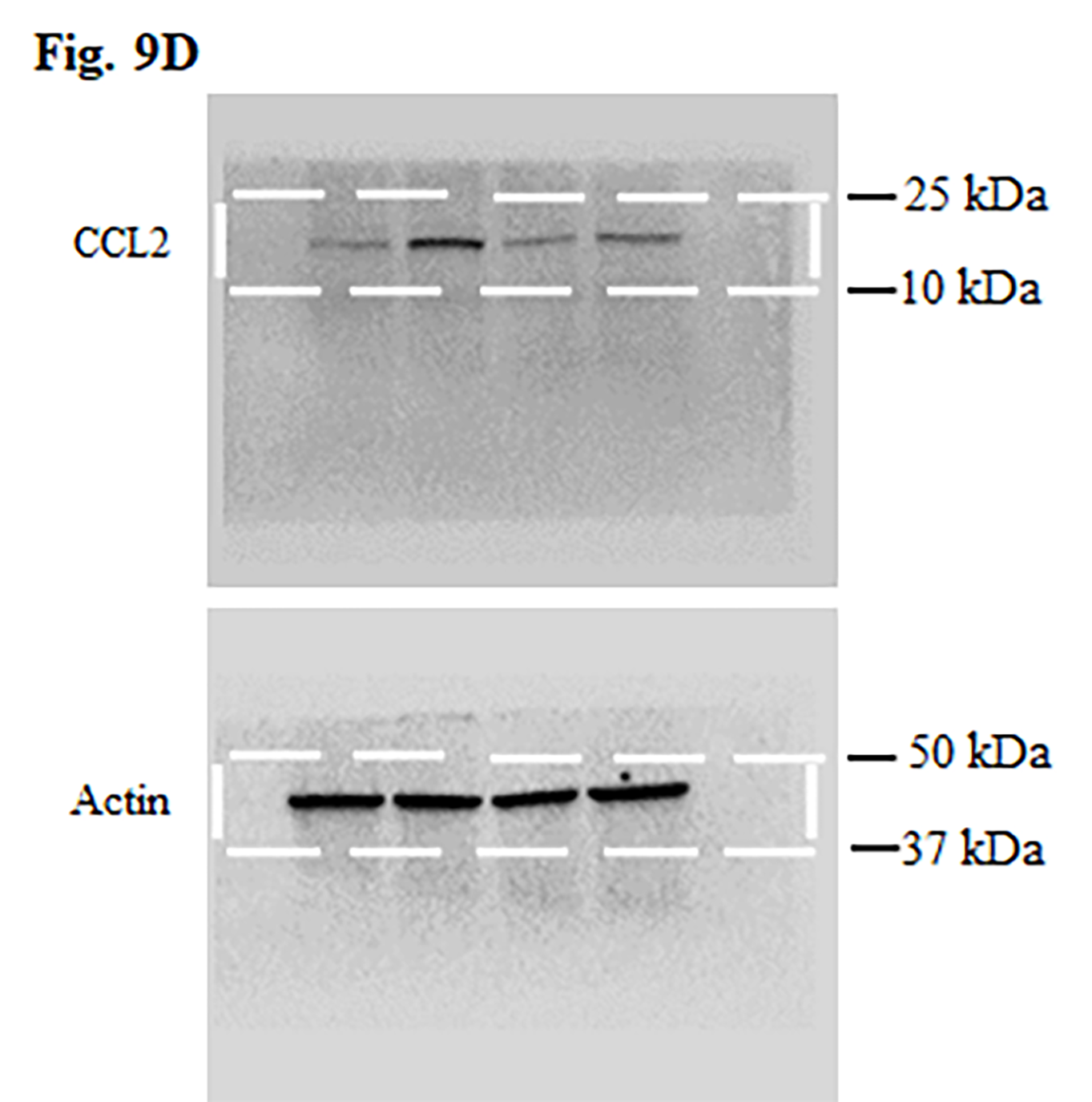

Supplement: Supplementary file 12 — Source data Fig. 9 [file 44319_2025_534_MOESM12_ESM.zip › Figure 9/9D.tif]

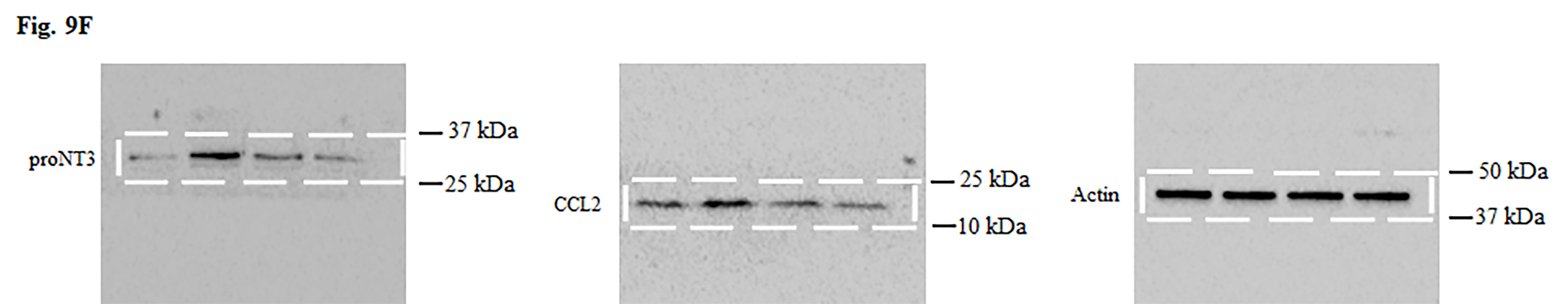

Supplement: Supplementary file 12 — Source data Fig. 9 [file 44319_2025_534_MOESM12_ESM.zip › Figure 9/Figure 9F.tif]

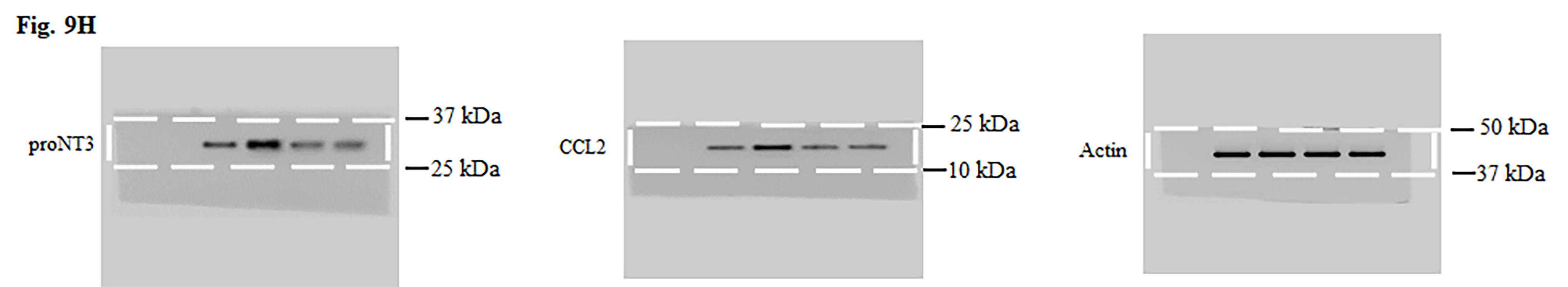

Supplement: Supplementary file 12 — Source data Fig. 9 [file 44319_2025_534_MOESM12_ESM.zip › Figure 9/Figure 9H.tif]

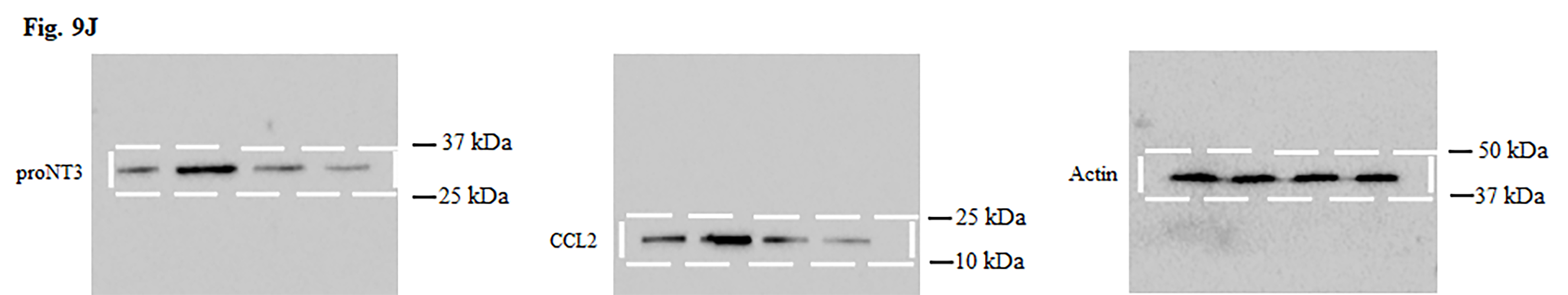

Supplement: Supplementary file 12 — Source data Fig. 9 [file 44319_2025_534_MOESM12_ESM.zip › Figure 9/Figure 9J.tif]

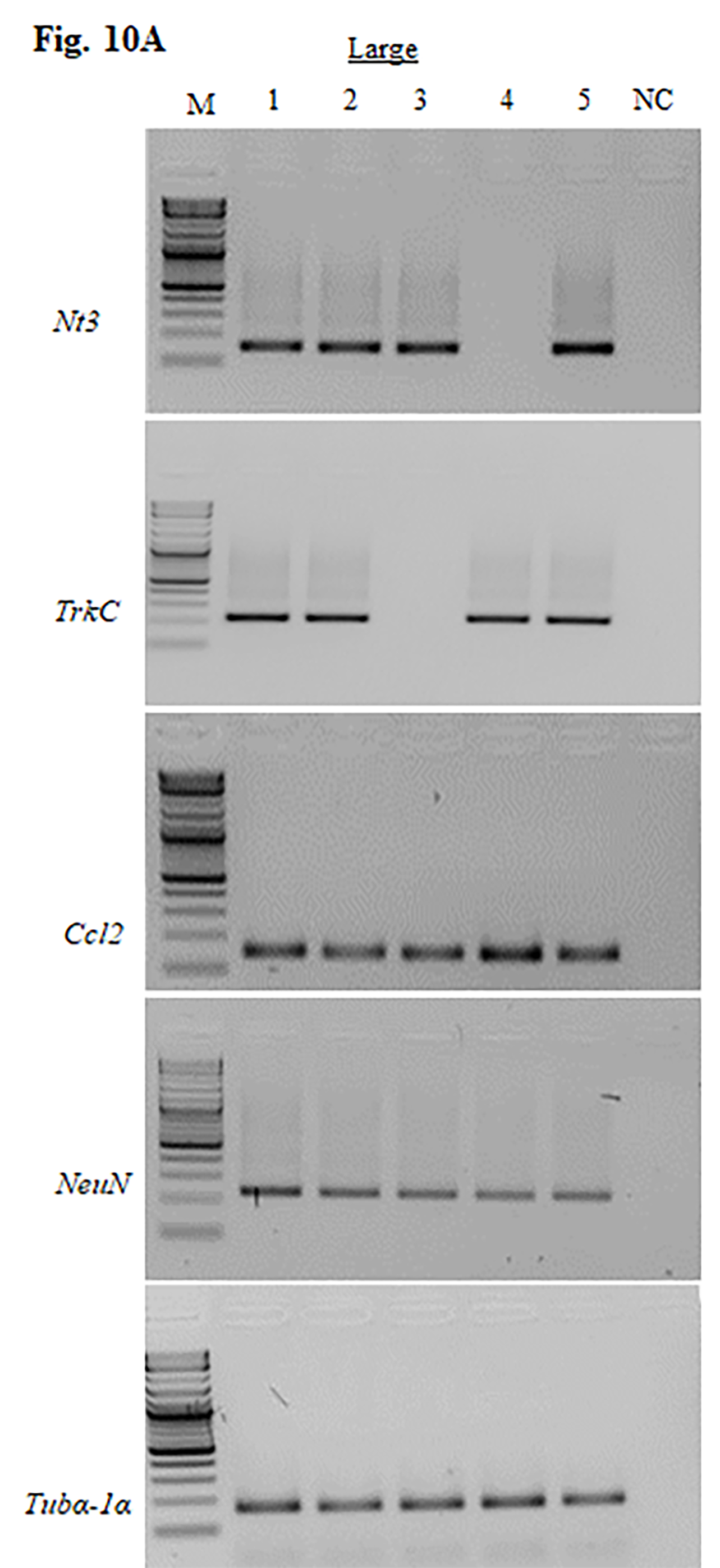

Supplement: Supplementary file 13 — Source data Fig. 10 [file 44319_2025_534_MOESM13_ESM.zip › Figure 10/10A.tif]

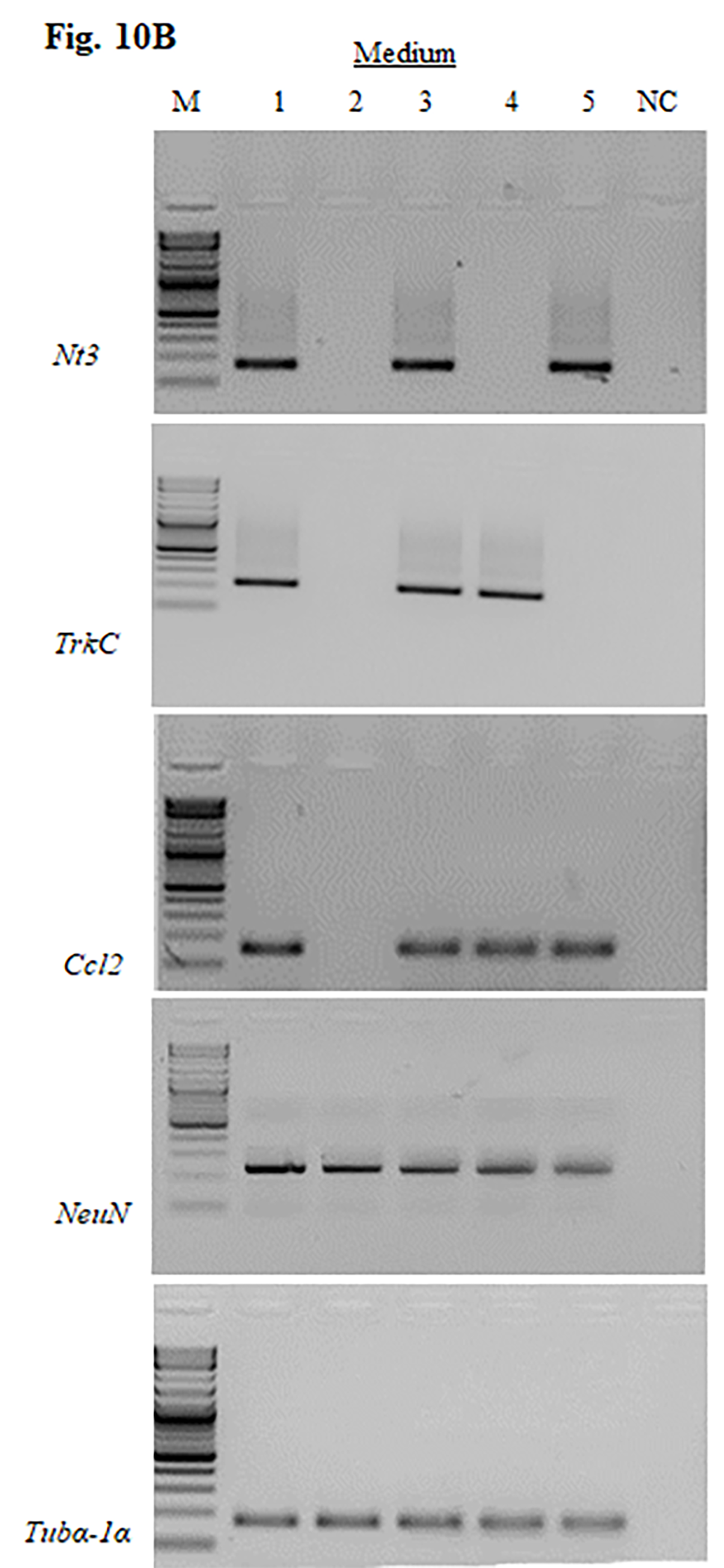

Supplement: Supplementary file 13 — Source data Fig. 10 [file 44319_2025_534_MOESM13_ESM.zip › Figure 10/10B.tif]

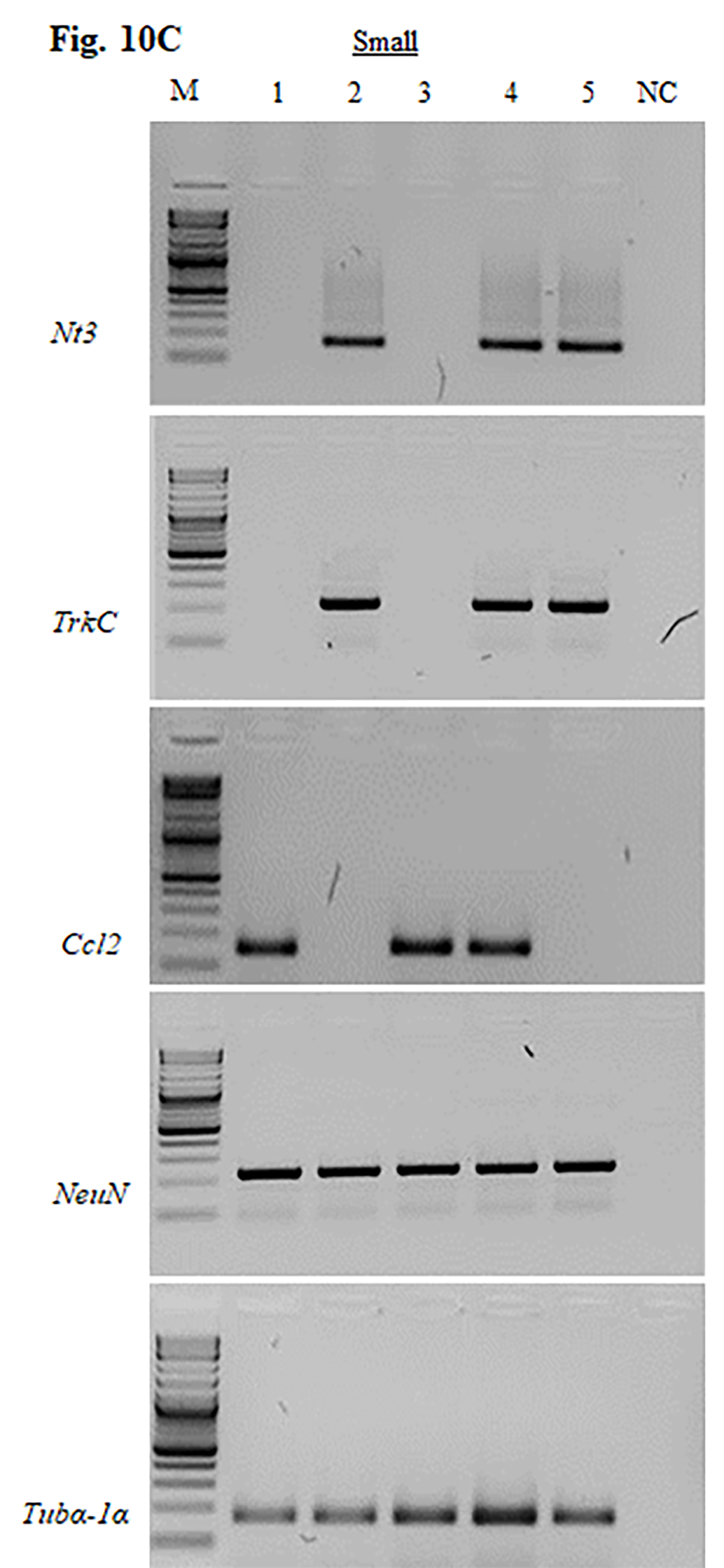

Supplement: Supplementary file 13 — Source data Fig. 10 [file 44319_2025_534_MOESM13_ESM.zip › Figure 10/10C.tif]

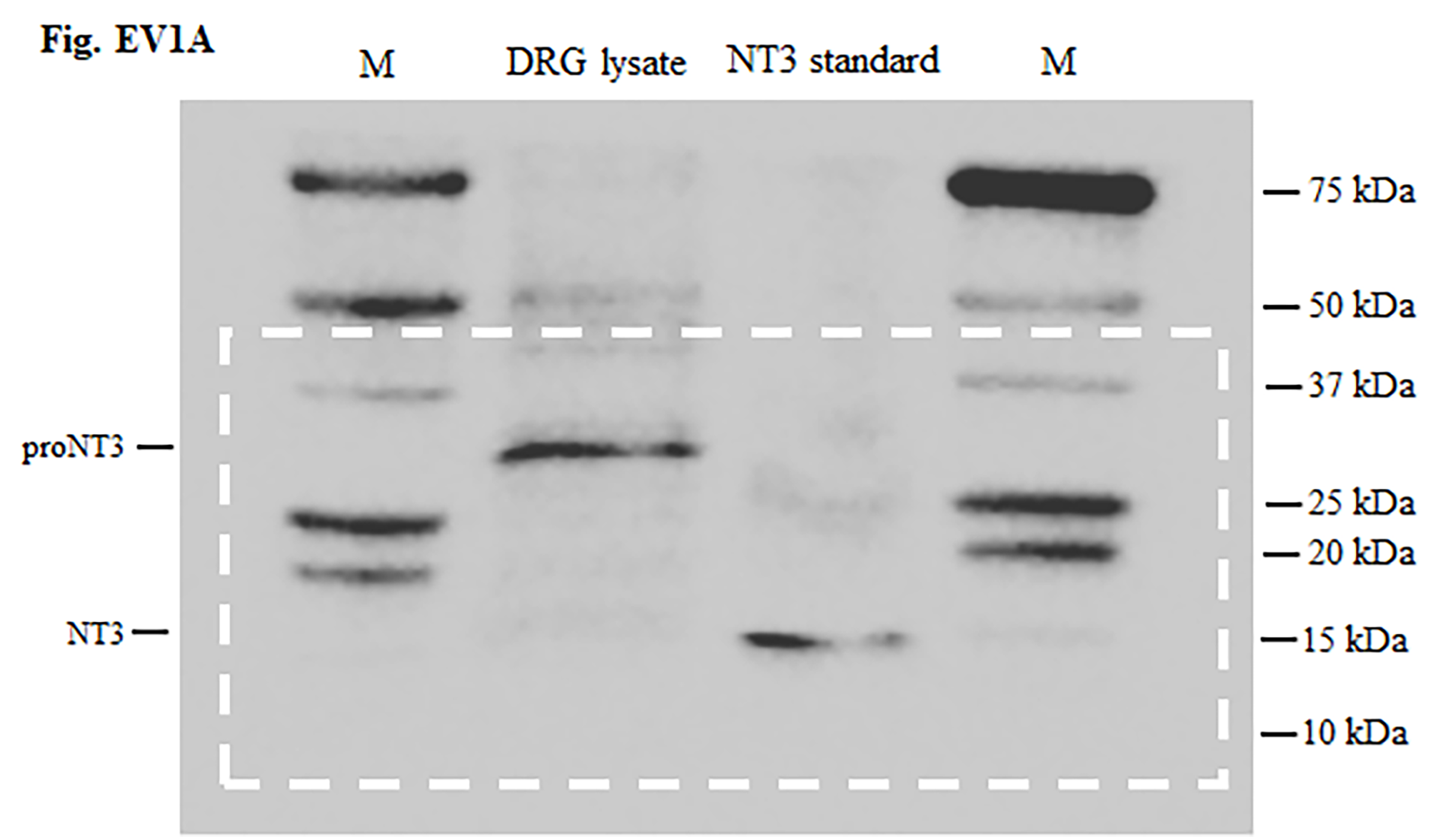

Supplement: Supplementary file 14 — Figure EV1 Source Data [file 44319_2025_534_MOESM14_ESM.zip › Figure EV1A 1B 1C/EV1A.tif]

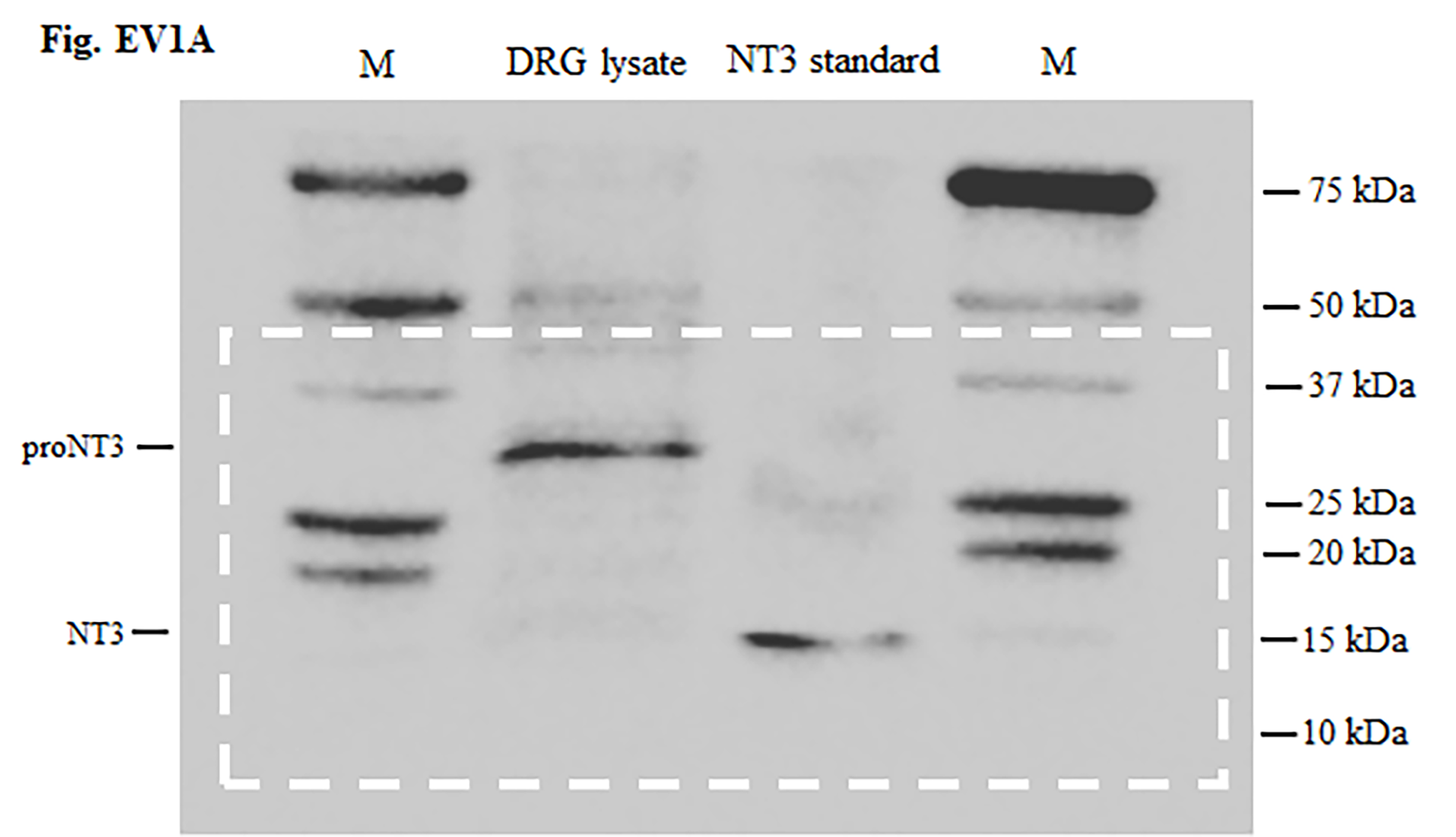

Supplement: Supplementary file 14 — Figure EV1 Source Data [file 44319_2025_534_MOESM14_ESM.zip › Figure EV1A 1B 1C/EV1B.tif]

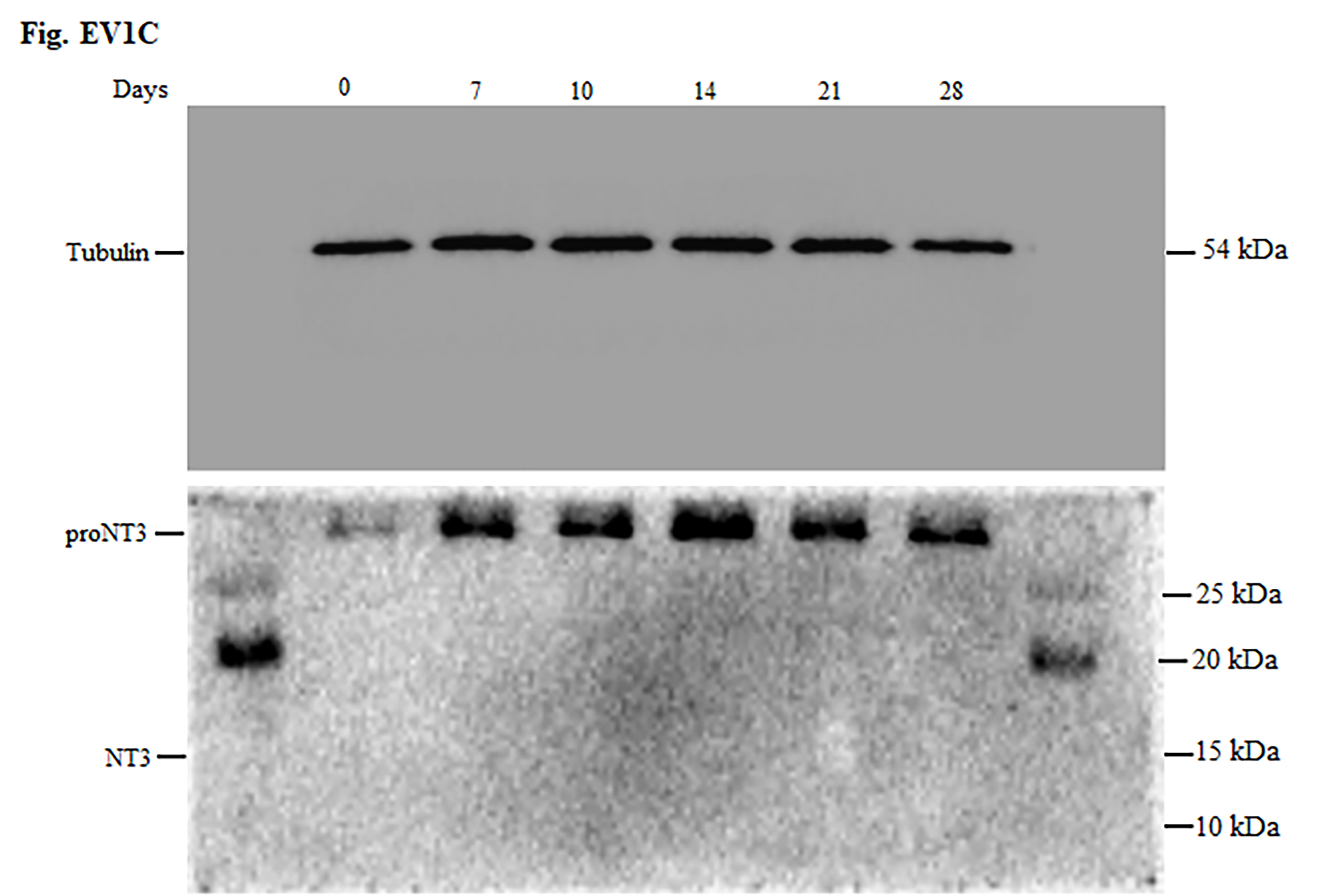

Supplement: Supplementary file 14 — Figure EV1 Source Data [file 44319_2025_534_MOESM14_ESM.zip › Figure EV1A 1B 1C/EV1C.tif]
