# Supplementary material for: Proneurotrophin-3 contributes to chemotherapy-induced neuropathic pain through TrkC-mediated CCL2 elevation in DRG neurons
Source: EMBO Rep. 2025 Nov 26;26(24):6141–58. doi: 10.1038/s44319-025-00534-1 (PMC12714783; doi:10.1038/s44319-025-00534-1)
Supplement: Supplementary file 15 — Expanded View Figures [file 44319_2025_534_MOESM15_ESM.pdf]

Expanded View Figures

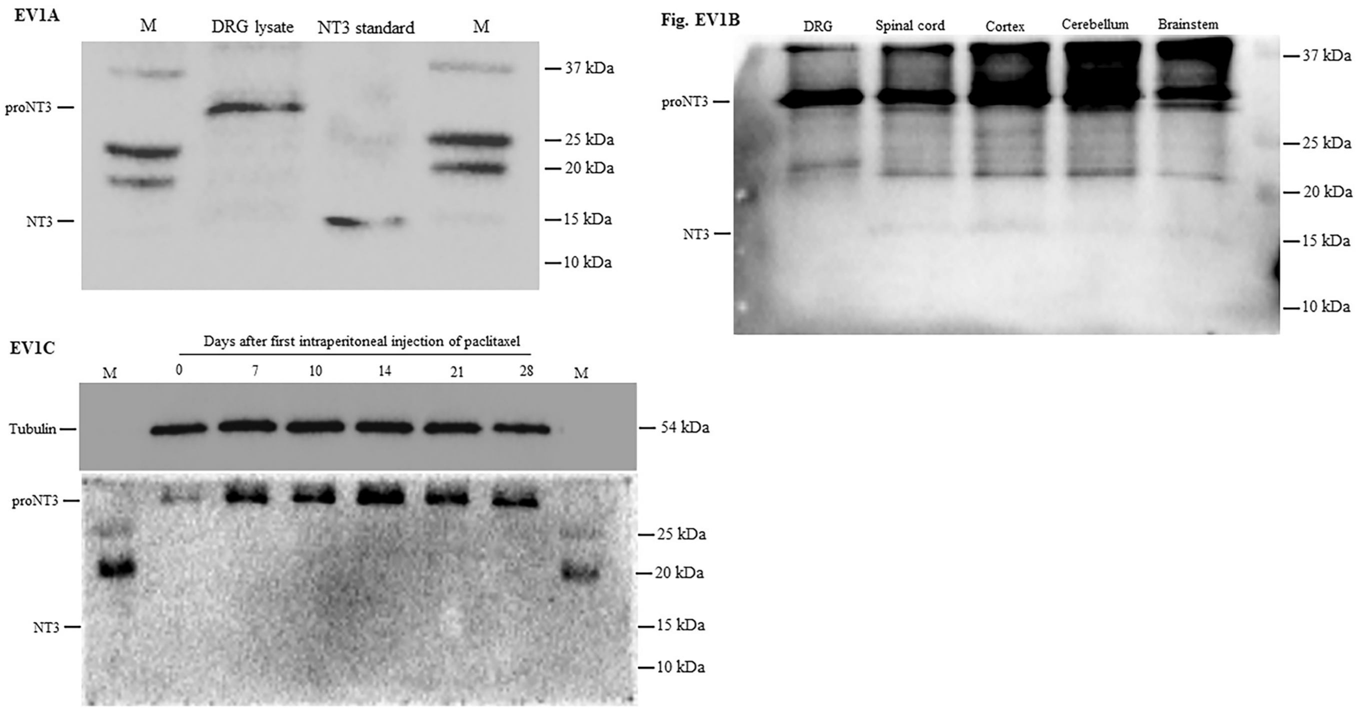

Figure EV1. Expression of proNT3, but not NT3, is detected in the DRG of naive and paclitaxel-treated mice.

(**A**) Total cellular lysates from the DRG of naive mice and recombinant NT3 standard (SinoBiological) are Western blotted with an anti-NT3 antibody (ANT-003, Alomone Labs) to demonstrate that this antibody recognizes both pro-NT3 and NT3. proNT3 (~32 kDa), but not NT3 (~ 14.5 kDa), is detected in DRG lysate from naive mice. (**B**) pro-NT3 is highly expressed in the DRG, spinal cord, cortex, cerebellum and brainstem. In contrast, NT3 is undetected in the DRG and expressed weakly in the spinal cord, cortex, cerebellum and brainstem. (**C**) Expression of proNT3 is time-dependently increased in the DRG after first intraperitoneal (i.p.) injection of paclitaxel. NT3 is not detected in the DRG on days 0, 7, 10, 14, 21, and 28 after the first i.p. paclitaxel injection. M: Molecular weight marker. Source data are available online for this figure.

 © The Author(s)
